# The Role of Chemotherapy Plus Immune Checkpoint Inhibitors in Oncogenic-Driven NSCLC: A University of California Lung Cancer Consortium Retrospective Study

**DOI:** 10.1016/j.jtocrr.2022.100427

**Published:** 2022-10-29

**Authors:** David J. Benjamin, Shuai Chen, Joanna B. Eldredge, Shiruyeh Schokrpur, Debory Li, Zhikuan Quan, Jason W. Chan, Amy L. Cummings, Megan E. Daly, Jonathan W. Goldman, Matthew A. Gubens, Jeremy P. Harris, Mark W. Onaitis, Viola W. Zhu, Sandip P. Patel, Karen Kelly

**Affiliations:** a Divsion of Hematology/Oncology, Department of Medicine, University of California Irvine, Irvine, California; b Division of Biostatistics, Department of Public Health Sciences, University of California Davis, Davis, California; c Division of Hematology and Oncology, Department of Medicine, University of California Davis School of Medicine, Sacramento, California; d Division of Hematology-Oncology, Department of Medicine, University of California, San Diego School of Medicine, La Jolla, California; e David Geffen School of Medicine, University of California Los Angeles, Los Angeles, California; f Department of Radiation Oncology, University of California, San Francisco, San Francisco, California; g Division of Hematology Oncology, Department of Medicine, David Geffen School of Medicine, University of California Los Angeles, Los Angeles, California; h Department of Radiation Oncology, University of California Davis School of Medicine, Sacramento, California; i Division of Hematology Oncology, Department of Medicine, University of California San Francisco, San Francisco, California; j Department of Radiation Oncology, University of California Irvine, Irvine, California; k Division of Cardiothoracic Surgery, Department of Surgery, University of California, San Diego, La Jolla, California; l Present Address: 1 Hoag Drive, Building 51, Newport Beach, California; m Present Address: Nuvalent, 1 Broadway, 14th Floor, Cambridge, Massachusetts; n Present Address: 999, 17th Street, Suite 200, Denver, Colorado

**Keywords:** Driver mutations, Oncogenic driven, Immune checkpoint inhibitors, Chemotherapy, Non–small cell lung cancer, Actionable mutations

## Abstract

**Introduction:**

There is a paucity of data on immune checkpoint inhibitors (ICIs) plus doublet chemotherapy (C) in patients with advanced lung cancer whose tumor harbors an actionable mutation. We sought to provide insight into the role of this combination in relation to chemotherapy alone in this patient population.

**Methods:**

We conducted a retrospective study at the five University of California National Cancer Institute–designated Comprehensive Cancer Centers. The primary end point was progression-free survival (PFS). Secondary end points included overall survival (OS) and significant adverse events. Adverse events in patients who received a tyrosine kinase inhibitor (TKI) post-ICI were also captured.

**Results:**

A total of 246 patients were identified, 170 treated with C plus ICI and 76 treated with C alone. Driver alterations included *EGFR* (54.9%), *KRAS* (32.9%), *ALK* (5.3%), *HER2*/*ERBB2* (2.9%), *ROS1* (1.2%), *MET* (1.2%), *RET* (0.8%), and *BRAF* non-V600 (0.8%). The overall PFS and OS hazard ratios were not significant at 1.12 (95% confidence interval 0.83–1.51; *p* = 0.472) and 0.86 (95% confidence interval: 0.60–1.24, *p* = 0.429), respectively. No significant differences in PFS or OS were observed in the mutational subgroups. Grade 3 or greater adverse events were lower in the C plus ICI group. The multivariate analysis for PFS and OS revealed a performance status (Eastern Cooperative Oncology Group) score of 2, and previous TKI treatment was associated with poorer outcomes with C plus ICI.

**Conclusions:**

Our study suggests that patients with oncogenic-driven NSCLC, primarily those with *EGFR*-driven tumors, treated with a TKI should not subsequently receive C plus ICI. Analysis from prospective clinical trials will provide additional information on the role of ICIs in this group of patients.

## Introduction

The treatment paradigm for patients with advanced NSCLC has dramatically changed in the past 10 years with the approval of targeted therapies and immune checkpoint inhibitors (ICIs) for those whose tumor harbors an actionable oncogenic driver alteration.^1^ Today, it is strongly recommended that patients with advanced lung adenocarcinoma undergo upfront next-generation sequencing testing for oncogenic driver mutations, including *EGFR*, *ALK*, *ROS1*, *MET*, *RET*, *BRAF*, *NTRK*, *KRAS*, and *HER2*/*ERBB2*, to determine eligibility for first-line treatment with efficacious tyrosine kinase inhibitors (TKIs). Despite the impressive benefit of these targeted therapies, most patients will experience disease progression and require chemotherapy. New classes of agents are needed to further prolong their survival.^2^ On the basis of preclinical data revealing antitumor activity for ICIs in *EGFR*-mutated tumor models, there was enthusiasm that this would translate into a clinical benefit for this group of patients.^3^ Three phase 3 clinical trials (KEYNOTE-010, OAK trial, and CheckMate 057) evaluating ICI monotherapy versus chemotherapy as a second-line treatment for lung cancer revealed no overall survival (OS) benefit in subgroup analysis of patients with *EGFR*-mutated NSCLC.^4^, ^5^, ^6^ A meta-analysis of five randomized clinical trials did not reveal improved survival with ICIs over chemotherapy in the subset of patients with *EGFR*-mutated NSCLC.^7^ Additional retrospective studies that included other actionable mutations such as *ALK* and *ROS1* also failed to reveal a clinical benefit with ICI.^8^, ^9^, ^10^, ^11^ The largest retrospective study known as IMMUNOTARGET, which evaluated 551 patients from 24 centers across 10 countries with *KRAS*, *EGFR*, *BRAF*, *MET*, *HER2*, *ALK*, *RET*, and *ROS1* molecular alterations, found limited efficacy with monotherapy ICI in patients with oncogenic-driven NSCLC.^12^ Similar findings have been observed across all stages of disease in NSCLC. In addition, there was no benefit with durvalumab consolidation (PACIFIC) in unresectable stage III NSCLC or with atezolizumab maintenance in high-risk, resected, early stage disease (IMpower010) in the cohorts of patients with *EGFR* or *ALK* genetic alterations.^13^^,^^14^

On the basis of these data, patients with *EGFR*- and *ALK*-mutated tumors were excluded from most randomized trials evaluating ICIs in combination with chemotherapy versus chemotherapy alone as frontline treatment for patients with advanced NSCLC. These trials evaluating ICI plus chemotherapy revealed a benefit for the triple drug combination, which is now the standard of care.^15^^,^^16^ The IMpower150 study is the only first-line trial to include patients with advanced NSCLC whose tumors harbor *EGFR* or *ALK* mutations.^17^ The study revealed that patients with *EGFR* or *ALK* tumor mutations did not benefit from chemotherapy plus atezolizumab compared with chemotherapy alone. Recently, Gadgeel et al.^18^ conducted a single-arm trial evaluating chemotherapy with ICI in *EGFR*- and *ALK*-mutated NSCLC previously treated with TKI, but the trial was halted owing to slow accrual. To our knowledge, there are no other retrospective or prospective data evaluating the efficacy of adding ICI to chemotherapy in oncogenic-driven NSCLC with actionable mutations.

Given the paucity of data evaluating ICI plus chemotherapy in patients with oncogenic mutated tumors, we sought to perform a retrospective study evaluating outcomes in patients with oncogenic-driven lung cancer treated with chemotherapy with or without ICI across the five National Cancer Institute–designated Comprehensive Cancer Centers (UC Davis, UC Irvine, UC Los Angeles, UC San Diego, UC San Francisco) in the University of California system.

## Materials and Methods

### Patients

The study schema is depicted in Supplementary Figure 1. Adult patients with oncogenic-driven lung cancer (*EGFR*, *ALK*, *ROS1*, *MET*, *RET*, *BRAF*, *NTRK*, *KRAS*, and *HER2*/*ERBB2*) detected by any Clinical Laboratory Improvement Amendments–approved laboratory test who were treated with chemotherapy with or without ICI were identified. Patients with SCLC transformation were excluded. Specifically, treatment regimens included (1) a platinum-based doublet regimen with or without bevacizumab, (2) a platinum-based doublet regimen with or without an ICI, or (3) a platinum-based doublet regimen with an ICI and bevacizumab. Anonymized data were collected for patients who met the inclusion criteria between January 2018 and December 2019 through the electronic medical record at each UC campus. The study was approved by the Institutional Review Board (IRB) of University of California, Davis. Through the University of California IRB Review Reliance/UC Memorandum of Understanding for IRB review of multicampus human subject research, three campuses (UC Irvine, UC San Diego, and UC San Francisco) relied on the UC Davis IRB review and approval of the study. The UCLA obtained separate IRB approval. Demographic data, TKI history, time on treatment, progression-free survival (PFS), OS, programmed death-ligand 1 (PD-L1) expression levels, grade 3 or greater toxicities, and subsequent treatment data were collected. For patients who received a TKI after an ICI, additional adverse event reporting was required.

### Study Objectives and End Points

The primary objective was to assess the PFS rate using the Response Evaluation Criteria in Solid Tumors version 1.1 as measured from the first administration of treatment to progression or death from any cause in patients whose tumor harbored an actionable oncogenic driver.^19^ Secondary objectives were OS from the first administration of treatment to death, to characterize grade 3 or greater treatment-related toxicities by the Common Terminology Criteria for Adverse Events 5.0 for patients receiving chemotherapy with or without ICI and in patients who received a TKI post-ICI therapy.

### Statistical Analysis

Statistics of all baseline demographics and patient characteristics were reported. Characteristics were compared between the two treatment groups (chemotherapy plus ICI versus chemotherapy alone) using Fisher’s exact tests for categorical variables and Wilcoxon ranked sum tests for continuous variables. Toxicities were summarized by descriptive statistics. Kaplan-Meier methods and the log-rank tests were used to compare unadjusted survival outcomes (OS and PFS) between the treatment groups. All patients had a minimum of 1 year of follow-up. Univariable and multivariable survival analyses were performed using Cox proportional hazards models. Factors with *p* value less than 0.1 in univariable analysis were included in multivariate analysis initially and further selected by backward method until all remained factors had *p* value less than 0.05 (except treatment which was always included because it was of primary interest). For this analysis, the mutational status was grouped into three categories with the additional assumption that group 1 (*EGFR*, *ALK*, *ROS1*, *HER2*, *RET*, and *MET*) was more likely to be never smokers compared with group 2 (*KRAS* G12C) and group 3 (*KRAS* non-G12C and *BRAF* non-V600E) who did not have an approved TKI therapy.^12^ To ensure the proportional hazards assumption holds, the assumption was evaluated using Schoenfeld residuals. To account for potential confounding and covariate imbalances between the treatment groups, we also used propensity score method as a sensitivity analysis.^20^ The propensity score was used to balance the covariate distribution between the groups. We estimated the probability of receiving chemotherapy plus ICI (the propensity score) for each patient according to relevant observed covariates by multivariable logistic regression. We evaluated the distribution of the propensity scores for each treatment group and confirmed sufficient overlap in the distributions to ensure that the groups were comparable. We then grouped the patients into quintiles according to their estimated propensity scores and used the Cochran-Mantel-Haenszel exact test and linear regression to verify that measured covariates were balanced across all strata. Propensity score-stratified Cox proportional hazards models were fitted to evaluate adjusted treatment effects in all patients and different patient characteristic subgroups, where strata were formed by quintiles of estimated propensity scores.

All analyses were performed using SAS 9.4 (SAS Institute Inc., Cary, NC) and R 4.0.4 (R Foundation for Statistical Computing, Vienna, Austria). All *p* values were two sided, and a *p* less than or equal to 0.05 was considered statistically significant.

## Results

### Patient Characteristics

A total of 246 patients were identified in the 2-year time frame. There were 170 patients treated with chemotherapy plus ICI and 76 patients treated with chemotherapy alone (Table 1). Bevacizumab was incorporated into the regimen in 32 patients, 29 patients in the chemotherapy plus ICI group and three patients in the chemotherapy-alone group. The mean age at treatment was 64.3 (range 30–89) years. Most patients were female (54.5%), White (55.6%), never smokers (55.3%), and with a performance status (PS) of 1 (60.6%). Adenocarcinoma was the predominant tumor histology (92.3%). Oncogenic driver mutations included *EGFR* mutations (54.9%), *KRAS* mutations (32.9%), *ALK* fusions (5.3%), *HER2*/*ERBB2* mutations (2.9%), *ROS1* fusion (1.2%), *MET* mutations (1.2%), *RET* fusions (0.8%), and *BRAF* non-V600E mutations (0.8%). No patient had a *BRAF* V600E mutation. Almost half of the patients did not receive a prior TKI. Approximately one-third of the patients had brain metastases. PD-L1 immunohistochemistry expression levels were similar across the two defined cohorts. There was a significant difference in treatment administration by mutational status. After adjusting for imbalances between the groups by propensity score method, we verified that measured covariates were balanced between the groups across all propensity score strata.

**Table 1:** Patient Characteristics

Characteristic	All (N = 246)	Chemotherapy + ICI (n = 170)	Chemotherapy (n = 76)	*p* Value	
	Mean (range)	Mean (range)	Mean (range)	Before propensity adjustment^a^	After propensity adjustment^b^
Age at treatment (range)	64.3 (30–89)	63.8 (30–87)	65.5 (31–89)	0.179	0.658
	N (%)	N (%)	N (%)		
Age				0.053	0.571
<65 y	114 (46.3)	86 (50.6)	28 (36.8)		
≥65 y	132 (53.7)	84 (49.4)	48 (63.2)		
Sex				0.891	1.000
Female	134 (54.5)	92 (54.1)	42 (55.3)		
Male	112 (45.5)	78 (45.9)	34 (44.7)		
Race				0.819	0.487
White	130 (55.6)	87 (54.7)	43 (57.3)		
Asian	77 (32.9)	53 (33.3)	24 (32.0)		
African American	7 (3.0)	6 (3.8)	1 (1.3)		
Other	20 (8.6)	13 (8.2)	7 (9.3)		
Histology				0.127	0.307
Adenocarcinoma	227 (92.3)	160 (94.1)	67 (88.2)		
Squamous	4 (1.6)	3 (1.8)	1 (1.3)		
Other	15 (6.1)	7 (4.1)	8 (10.5)		
Smoking				0.287	0.223
Current	10 (4.1)	9 (5.3)	1 (1.3)		
Former	100 (40.7)	66 (38.8)	34 (44.7)		
Never	136 (55.3)	95 (55.9)	41 (54.0)		
ECOG Performance status				0.226	0.173
0	67 (27.8)	51 (30.9)	16 (21.1)		
1	146 (60.6)	96 (58.2)	50 (65.8)		
2	25 (10.4)	17 (10.3)	8 (10.5)		
3	3 (1.2)	1 (0.6)	2 (2.6)		
Oncogenic mutation				0.040	0.587
*ALK* fusion	13 (5.3)	7 (4.1)	6 (7.9)		
*BRAF* mutations (nonV600E)	2 (0.8)	1 (0.6)	1 (1.3)		
*EGFR* mutations	135 (54.9)	86 (50.6)	49 (64.5)		
*HER2* mutations	7 (2.9)	5 (2.9)	2 (2.6)		
*KRAS* mutations	81 (32.9)	66 (38.8)	15 (19.7)		
*MET* mutations	3 (1.2)	2 (1.2)	1 (1.3)		
*RET* fusion	2 (0.8)	2 (1.2)	0 (0.0)		
*ROS1* fusion	3 (1.2)	1 (0.6)	2 (2.6)		
Prior number of TKIs				0.282	0.673
0	118 (48.0)	88 (51.8)	30 (39.5)		
1	52 (21.1)	34 (20.0)	18 (23.7)		
2	54 (22.0)	36 (21.2)	18 (23.7)		
3	19 (7.7)	10 (5.9)	9 (11.8)		
≥4	3 (1.2)	2 (1.2)	1 (1.3)		
Brain metastases				0.776	0.586
No	153 (62.2)	107 (62.9)	46 (60.5)		
	
Yes	93 (37.8)	63 (37.1)	30 (39.5)		
PD-L1 expression				0.670	0.941
0	76 (39.8)	53 (37.9)	23 (45.1)		
1–49	63 (33.0)	48 (34.2)	15 (29.4)		
50+	52 (27.2)	39 (27.9)	13 (25.5)		

ECOG, Eastern Cooperative Oncology Group; ICI, immune checkpoint inhibitor; PD-L1, programmed death-ligand 1; TKI, tyrosine kinase inhibitor.

a *p* values were from Fisher’s exact tests for categorical variables and Wilcoxon ranked sum tests for continuous variable.

b Patients were grouped into quintiles according to their estimated propensity scores and used Cochran-Mantel-Haenszel exact tests for categorical variables and linear regression for continuous variable to verify that covariates were balanced across all strata.

### Clinical Outcomes

**Figure 1:**
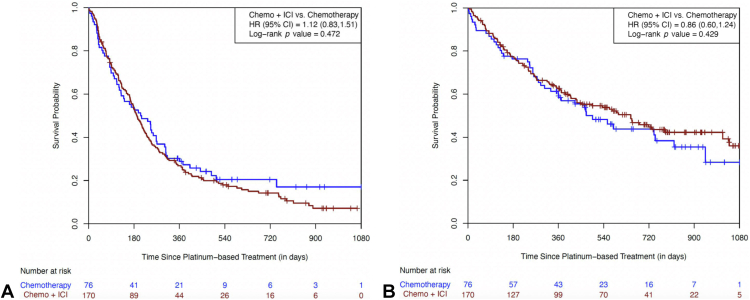
(*A*) Progression-free survival in the study cohort. (*B*) Overall survival in the study cohort. Chemo, chemotherapy; CI, confidence interval; HR, hazard ratio; ICI, immune checkpoint inhibitor.

The median (range) follow-up time was 193 (0–1096) days for PFS and 418 (2–1402) days for OS. For all patients treated with ICI and chemotherapy versus chemotherapy alone, the PFS hazard ratio (HR) was not significant at 1.12 (95% confidence interval [CI]: 0.83–1.51, *p* = 0.472) (Fig. 1*A*). The median PFS was 192 days (95% CI: 165–222 d) for the chemotherapy plus ICI cohort and 208 days (95% CI: 122–268 d) for the chemotherapy cohort. The OS HR was 0.86 (95% CI: 0.60–1.24, *p* = 0.429) (Fig. 1*B*). with a median OS of 648 days (95% CI: 430–1013 d) among the ICI and chemotherapy cohort and 496 days (95% CI: 330–820 d) among the chemotherapy-alone cohort.

**Figure 2:**
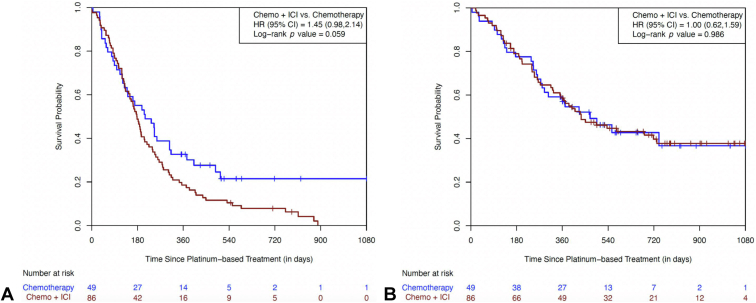
(*A*) Progression-free survival in the *EGFR* subgroup. (*B*) Overall survival in the *EGFR* subgroup. Chemo, chemotherapy; CI, confidence interval; HR, hazard ratio; ICI, immune checkpoint inhibitor.

Most patients in this study had tumors with *EGFR* mutations (N = 135). The PFS trended toward an inferior survival with the addition of an ICI with a HR of 1.45 (95% CI: 0.98–2.14, *p* = 0.059). The median PFS was 178 days (95% CI: 145–206 d) with the combination and 210 days (95% CI: 128–306 d) with chemotherapy alone (Fig. 2*A*). The OS HR was 1.00 (95% CI: 0.62–1.59, *p* = 0.986) with similar median OS times of 434 days (95% CI: 325–732 d) for the ICI cohort and 469 days (95% CI: 281 d–not reached [NR]) for the chemotherapy cohort (Fig. 2*B*).

**Figure 3:**
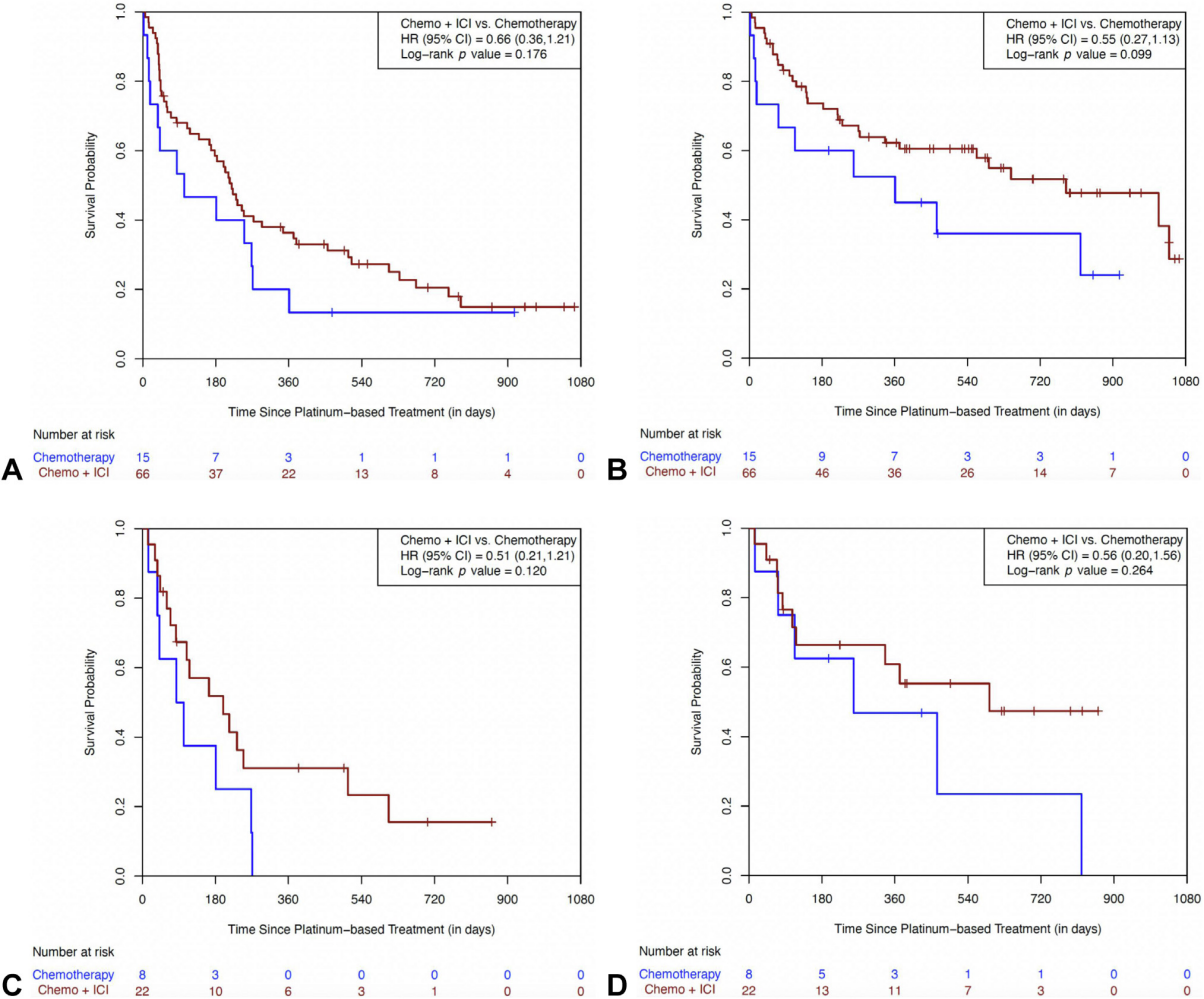
(*A*) Progression-free survival in the *KRAS* subgroup. (*B*) Overall survival in the *KRAS* subgroup. (*C*) Progression-free survival in the *KRAS* G12C subgroup. (*D*) Overall survival in the *KRAS* G12C subgroup. Chemo, chemotherapy; CI, confidence interval; HR, hazard ratio; ICI, immune checkpoint inhibitor.

The *KRAS*-mutated tumors were identified in 81 patients. Outcomes were numerically favorable for both PFS (HR = 0.66 [95% CI: 0.36–1.21, *p* = 0.176]) and OS (HR = 0.55 [95% CI: 0.27–1.13, *p* = 0.099]) for patients receiving chemotherapy plus ICI (Fig. 3*A* and *B*). The median PFS and OS were 219 days (95% CI: 164–294 d) and 784 days (95% CI: 336 d–NR) for the ICI-treated patients and 102 days (95% CI: 15–268 d) and 361 days (95% CI: 15 d–NR) for the patients administered with chemotherapy alone, respectively. In the subset of patients whose tumor harbored a *KRAS* G12C mutation (N = 30, 37% of all KRAS tumors), encouraging outcomes were also observed for the addition of ICI to chemotherapy with a PFS HR of 0.51 (95% CI: 0.21–1.21; *p* = 0.120) and an OS HR of 0.56 (95% CI: 0.20–1.56; *p* = 0.264) (Fig. 3*C* and *D*). The median PFS was 199 days (95% CI: 69–507 d) in those treated with ICI and chemotherapy compared with 93 days (95% CI: 15–268 d) for those treated with chemotherapy alone. The median OS for those receiving ICI plus chemotherapy was 593 days (95% CI: 107 d–NR) compared with 258 days (95% CI: 15–820 d) for those receiving chemotherapy alone.

**Figure 4:**
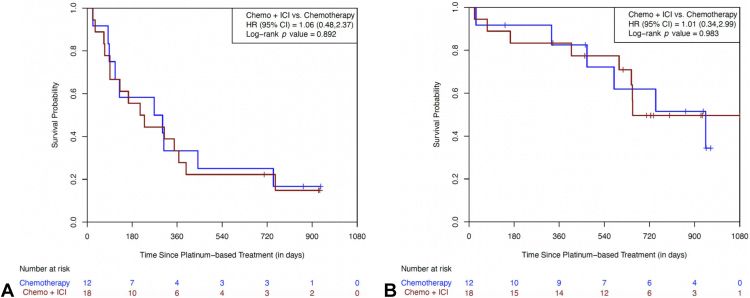
(*A*) Progression-free survival in other actionable mutations. (*B*) Overall survival in other actionable mutations. Chemo, chemotherapy; CI, confidence interval; HR, hazard ratio; ICI, immune checkpoint inhibitor.

There were 30 patients who had a variety of other actionable mutations in their tumors, including 13 *ALK* fusions, seven *HER2* mutations, three *MET* mutations, three *ROS-1* fusions, two *BRAF* non-V600 mutations, and two *RET* fusions. The three patients with *MET*-altered tumors (two with mutations and one with amplification) were all never smokers. The PFS HR for this group of patients was 1.06 (95% CI: 0.48–2.37; *p* = 0.892) (Fig. 4*A*). Median PFS was 221 days for patients treated with chemotherapy and ICI and 285 days for patients treated with doublet chemotherapy. In addition, the OS HR was 1.01 (95% CI: 0.34–2.99; *p* = 0.983) with a median OS of 653 days (95% CI: 599–NR) for the ICI cohort and 944 days (95% CI: 330–NR) for the chemotherapy-alone cohort (Fig. 4*B*).

Additional subgroup analyses by smoking history, PD-L1 expression levels, and TKI status did not reveal significant PFS differences (Supplementary Table 1 and Supplementary Fig. 2). The PFS HRs were greater than 1 for never smokers, TKI-naive or TKI-treated patients, and those whose tumors did not express PD-L1 or had a PD-L1 of greater than 50%. Smokers and patients with PD-L1 expression levels between 1% and 49% had a PFS HR less than 1. The OS differences were nonsignificant with the exception of a beneficial outcome for the immunotherapy combination in patients with tumor PD-L1 expression scores between 1 and 49 (HR = 0.41 [95% CI: 0.20–0.83, *p* = 0.010]) (Supplementary Table 1 and Supplementary Fig. 3). Nevertheless, the significance in PD-L1 expression scores between 1 and 49 was not retained after adjustment for covariates using propensity score method (Supplementary Table 2). The OS HR was greater than 1 for TKI-naive patients and less than 1 regardless of smoking history, previous TKI use, and in patients with PD-L1–expressing tumors of 0%. Patients with PD-L1–expressing tumors of greater than or equal to 50% had an OS HR of 1.00.

In the univariate analysis for PFS, significant variables defined as having a *p* value of less than 0.1 were age above or equal to 65 years, female sex, Asian race, PS 2 (versus 0–1), PD-L1 expression of greater than or equal to 50%, and treatment with a TKI (Table 2). After adjusting for these covariates in the multivariate analysis, PS 2 and prior TKI treatment retained their poor prognostic significance (Table 2). For OS, poor prognosis was significantly associated with age above or equal to 65 years, PS 2, and treatment with a TKI in the univariate analysis, and all three factors retained their significance in the multivariate analysis (Table 3). A sensitivity analysis was conducted in all patients and subgroups using a propensity score method to adjust for covariate imbalance. There was no significant difference in PFS or OS between the treatment groups or in any of the subgroups except for a significant detrimental effect on PFS in the *EGFR* subset treated with the combination of chemotherapy and ICI (HR = 1.67 [95% CI: 1.00–2.80, *p* = 0.049]) (Supplementary Table 2).

**Table 2:** Univariable and Multivariable Survival Analysis for PFS Using Cox proportional Hazards Models

Patient Characteristics	Univariable			Multivariable		
	HR	(95% CI)	*p* Value	HR	(95% CI)	*p* Value
Treatment						
Chemotherapy	1.00			1.00		
Chemotherapy + ICI	1.12	0.83–1.51	0.473	1.10	0.82–1.50	0.520
Age, y						
<65	1.00					
≥65	1.34	1.02–1.76	0.038			
Sex						
Male	1.00					
Female	0.78	0.59–1.02	0.069			
Race						
Non-Asian	1.00					
Asian	1.32	0.98–1.78	0.064			
Smoke						
No	1.00					
Yes	1.03	0.78–1.35	0.839			
ECOG performance status						
0–1	1.00			1.00		
2+	2.50	1.66–3.77	<0.001	2.25	1.48–3.42	<0.001
Histology						
Adenocarcinoma	1.00					
Other	0.67	0.37–1.19	0.172			
Prior number of TKIs						
0	1.00			1.00		
1+	1.76	1.33–2.33	<0.001	1.65	1.24–2.20	0.001
Brain metastases						
No	1.00					
Yes	1.04	0.79–1.37	0.787			
Oncogenic mutation						
(1) *EGFR*, *ALK*, *RET*, *ROS1*, *HER2*, *MET*	1.00					
(2) *KRAS* G12C	1.14	0.74–1.76	0.548			
(3) *KRAS* non-G12C, *BRAF* non-V600E	0.77	0.55–1.09	0.144			
PD-L1 expression levels						
0	1					
1–49	0.93	0.65–1.32	0.670			
50+	0.64	0.43–0.96	0.029			
Bevacizumab						
No	1.00					
Yes	1.15	0.78–1.70	0.474			

*Note:* Variables with *p* value less than 0.1 in univariate analyses were included in multivariate analysis initially and further selected by backward method. Treatment variable was always included.

CI, confidence interval; ECOG, Eastern Cooperative Oncology Group; HR, hazard ratio; ICI, immune checkpoint inhibitor; PD-L1, programmed death-ligand 1; PFS, progression-free survival; TKI, tyrosine kinase inhibitor.

**Table 3:** Univariable and Multivariable Survival Analysis for Overall Survival Using Cox Proportional Hazards Models

Patient Characteristics	Univariable			Multivariable		
	HR	(95% CI)	*p* Value	HR	(95% CI)	*p* Value
Treatment						
Chemotherapy	1.00			1.00		
Chemotherapy + ICI	0.86	0.60–1.24	0.430	0.91	0.63–1.32	0.621
Age						
<65 y	1.00			1.00		
≥65 y	1.67	1.18–2.38	0.004	1.54	1.07–2.22	0.019
Sex						
Male	1.00					
Female	0.77	0.55–1.09	0.142			
Race						
Non-Asian	1.00					
Asian	1.00	0.69–1.46	0.993			
Smoke						
No	1.00					
Yes	0.97	0.69–1.37	0.869			
ECOG performance status						
0–1	1.00			1.00		
2+	2.71	1.72–4.26	<0.001	2.41	1.51–3.83	<0.001
Histology						
Adenocarcinoma	1.00					
Other	0.80	0.39–1.63	0.531			
Prior number of TKIs						
0	1.00			1.00		
1+	1.65	1.16–2.34	0.005	1.60	1.11–2.30	0.011
Brain metastases						
No	1.00					
Yes	0.85	0.60–1.22	0.386			
Oncogenic mutation						
(1) *EGFR*, *ALK*, *RET*, *ROS1*, *HER2*, *MET*	1.00					
(2) *KRAS* G12C	1.28	0.75–2.18	0.363			
(3) *KRAS* non-G12C, *BRAF* non-V600E	0.90	0.58–1.39	0.629			
PD-L1 expression levels						
0	1					
1–49	0.93	0.60–1.45	0.744			
50+	0.72	0.44–1.18	0.195			
Bevacizumab						
No	1.00					
Yes	1.21	0.75–1.95	0.432			

*Note:* Variables with *p* value less than 0.1 in univariate analyses were included in multivariate analysis initially and further selected by backward method. Treatment variable was always included.

CI, confidence interval; ECOG, Eastern Cooperative Oncology Group; HR, hazard ratio; ICI, immune checkpoint inhibitor; PD-L1, programmed death-ligand 1; TKI, tyrosine kinase inhibitor.

Grade 3 or greater adverse events were collected. Overall, there were more significant adverse events observed in the chemotherapy cohort (47%) as compared with the chemotherapy plus ICI cohort (17%) (Supplementary Table 3). This was due to cytopenia with 18 events occurring in patients receiving chemotherapy versus nine events observed in patients receiving chemotherapy plus ICI. There were seven (3.9%) inflammatory adverse events (mucositis—1, cellulitis—1, myocarditis—1, pneumonitis—2, transaminitis—1, and nephritis—1) observed in patients receiving the combination and three (4.2%) events in the chemotherapy cohort (mucositis—2 and cellulitis—1). Other adverse events of interest such as rash (N = 3) and hyperthyroidism (N = 1) were only observed in the chemotherapy plus ICI group. Only one patient who received chemotherapy, bevacizumab, and atezolizumab had a possible grade 5 treatment-related death. Seven of 68 patients (10.3%) who received a TKI as their next line of treatment after an ICI had a grade 3 to 4 adverse event (Supplementary Table 4). Transaminitis was the most frequent AE. Significant AEs developed within 30 days of starting a TKI in three patients. One additional patient developed an AE within 60 days. Three patients developed a significant toxicity greater than 100 days after TKI administration.

## Discussion

This multi-institutional retrospective trial revealed that there was no PFS or OS benefit to adding an ICI to chemotherapy for patients with oncogenic-driven lung cancer. Nevertheless, in specific driver subsets, interesting trends emerged that are consistent with ICI monotherapy data reported from the IMMUNOTARGET study.^12^ In the largest cohort of patients with *EGFR*-mutated tumors, our finding is supported by a small, single institutional retrospective analysis evaluating OS. After progression on osimertinib, 29 patients received platinum-based chemotherapy and 12 patients received pemetrexed, carboplatin, and pembrolizumab.^21^ The OS HRs were 1.45 (95% CI: 0.71–2.96) unadjusted and 2.31 (95% CI: 0.87–6.15) adjusted. There was no PFS or OS advantage for 79 patients with *EGFR*-mutated tumors in the exploratory analysis of IMpower150 comparing paclitaxel, carboplatin, and bevacizumab (BCP) to paclitaxel, carboplatin, and atezolizumab.^22^^,^^23^ Nevertheless, the exploratory analysis of 89 patients receiving BCP or ABCP (chemotherapy, bevacizumab, and atezolizumab) trended in a favorable direction for the addition of atezolizumab.^22^^,^^23^ The definitive role of adding an ICI to a standard chemotherapy-based regimen in patients with an *EGFR*-mutated tumor awaits the results from several randomized trials (the phase 3 CheckMate 722 [NCT02864251] comparing chemotherapy plus nivolumab with chemotherapy alone, KEYNOTE 789 [NCT03515837] comparing pemetrexed plus a platinum with or without pembrolizumab, and the randomized phase 2 trial TH-138 [NCT03786692] evaluating the three-drug regimen of pemetrexed, carboplatin, and bevacizumab to the four-drug regimen of pemetrexed, carboplatin, bevacizumab, and atezolizumab).

The favorable survival in the *KRAS*-mutant population with chemotherapy plus ICI aligns with other data reported. A descriptive post hoc analysis from KEYNOTE 189 identified 89 patients with a *KRAS*-mutated tumor. There were 59 patients treated with chemotherapy plus pembrolizumab and 30 patients who received chemotherapy alone. A beneficial trend in PFS was found for patients receiving the addition of pembrolizumab with a PFS HR of 0.47 (95% CI: 0.29–0.77), but no OS advantage was observed with HR of 0.79 (95% CI: 0.45–1.38).^24^ A total of 225 patients enrolled in the IMpower150 trial had a *KRAS* mutation in their tumor. In the comparison between chemotherapy plus bevacizumab or atezolizumab (N = 145), OS but not PFS favored the addition of atezolizumab whereas the four-drug regimen prolonged both PFS and OS (N = 151) compared with chemotherapy and bevacizumab.^25^

Numerically favorable results for chemotherapy plus ICI were found in the *KRAS* G12C subset. Similar findings were observed for PFS in the small subset of patients with *KRAS* G12C-mutated lung cancer (N = 37) in KEYNOTE 189, but, in contrast to our study, no promising OS benefit was observed for the ICI combination.^24^ This difference may be due to the small sample sizes of both studies but raises the question regarding emerging data suggesting that other factors such as PD-L1 expression level, TMB, STK11, and KEAP1 may influence ICI outcomes in patients with KRAS-mutated tumors, none of which were accounted for in these two studies. STK11 and KEAP1 comutations have been associated with worse outcomes in patients with *KRAS*-mutant lung cancer treated with an ICI. The IMpower150 post hoc analysis of patients with *KRAS*-mutated tumors included STK11 and KEAP1 status. There were 101 patients who had tumors with *STK11* mutation and *KEAP1* mutations or both mutations. There was no survival advantage for paclitaxel, carboplatin, and atezolizumab, but patients receiving ABCP had a PFS HR of 0.49 (95% CI: 0.28–0.84) and an OS HR of 0.60 (95% CI: 0.34–1.030). Further elucidating the role of ABCP in this ICI-resistant co-mutated subset is warranted.

The low incidence of other actionable mutations was surprising. In comparison to the larger IMMUNOTARGET registry, we had a similar incidence of *ALK* and *ROS1* alterations but a much lower incidence of *MET*, *HER2*/*ERBB2*, and *RET* genetic abnormalities, and no patient had a *BRAF* V600E mutation. Antecedent use of chemotherapy plus ICI or ICI monotherapy in this more rare subset is a potential reason patients with *BRAF*-mutated NSCLC did not receive post-targeted chemoimmunotherapy. Owing to small numbers, patients with other driver mutated tumors were consolidated into one group for analysis. Despite the small numbers and mutational heterogeneity, there was no benefit to the addition of ICI to chemotherapy for PFS or OS. Although these data are inconclusive, the results are consistent with the lack of benefit observed in our *EGFR*-mutated population and the lack of benefit found with ICI monotherapy in the IMMUNOTARGET registry.^12^ There are no published data on the role of chemotherapy plus an ICI compared with chemotherapy alone in this group of patients. The IMpower150 enrolled a total of 34 patients with *ALK*-positive tumors, but the results were not reported separately. It is unlikely that a randomized trial would be conducted comparing chemotherapy with or without an ICI, but additional real-world data would be instrumental in addressing this frequently asked question. This question is particularly pertinent in determining the optimal treatment sequence for patients with *BRAF* V600E and *MET*-positive tumors where both TKIs and ICI-based regimes are considered appropriate in the frontline setting.^26^

Survival curves by smoking status, line of TKI treatment, or PD-L1 expression levels did not reveal a PFS or OS benefit for chemotherapy plus ICI, except for patients whose tumor had PD-L1 expression scores between one and 49, who had a favorable OS prolongation with the addition of ICI. In this subset, 60% of the patients had *EGFR*-mutated tumors and 38% had *KRAS*-mutated tumors. This result is difficult to explain but may be spurious given the observed OS-associated benefit but lack of PFS-associated benefit and lack of OS or PFS benefit in the propensity score-matched analysis.

To account for imbalances in the covariates, we conducted additional analyses. In the univariate and multivariate analyses, PS 2 and prior TKI treatment were identified as independent negative predictors for PFS and OS benefit with chemotherapy and ICI. In addition, PS 2 is a well-known poor prognostic factor. We are not aware of data evaluating patients with PS 2 receiving chemotherapy plus ICI. The available data with ICI monotherapy reveal mixed results. CheckMate 171 evaluated nivolumab in 811 previously treated patients with squamous histology. In the subset of 103 patients with PS 2, outcome was lower than the overall populations.^27^ Similar results were found in the nivolumab-expanded access trial CheckMate 169.^28^ The PeP2 study was a single-arm, phase 2 study that evaluated pembrolizumab in 60 patients with PS 2. The authors concluded that OS was similar to historical data from KEYNOTE-001 that enrolled patients with PS 0 to 1.^29^ There is a need to conduct randomized trials to clearly determine the role of ICI alone versus ICI with chemotherapy in patients with PS 2 regardless of tumor mutational status. The finding that TKI administration is a poor prognostic factor for ICI plus chemotherapy supports our hypothesis. Oncogenic-driven tumors for which a TKI was routinely available as the initial treatment included *EGFR*, *ALK*, and *ROS1*. These tumors have been characterized as weakly immunogenetic by frequently having no or low tumor PD-L1 expression, low tumor mutational burden, and low numbers of tumor-infiltrating lymphocytes cultivating in an immunosuppressive tumor microenvironment.^30^ Data from KEYNOTE 189 (KN-189) revealing a benefit for the combination of chemotherapy plus pembrolizumab in patients with no or low PD-L1 expression suggested that chemotherapy could activate an antitumor immune response by release of neoantigens and cytokines in weakly immunogenic tumors. Thus, it is reasonable to evaluate chemotherapy plus ICI in oncogenic tumors. Although the definitive answer about the role of ICI plus chemotherapy awaits the results of randomized clinical trials, results from our study and IMpower150 did not reveal an efficacy advantage for chemotherapy plus ICI. In our study, age above or equal to 65 years was not an independent negative predictor of ICI benefit for PFS, but it was for OS. The influence of age on treatment with chemotherapy plus ICI in this population is unknown and awaits the results from randomized trials.

Our study revealed that chemotherapy plus an ICI was well tolerated with an overall lower number of significant AEs compared with chemotherapy. The higher toxicity rate with chemotherapy alone was not expected. Detailed dosing data were not collected, but patients in the chemotherapy cohort were older and previously treated compared with the ICI cohort. Another explanation could be that chemotherapy doses were lower in the chemotherapy plus ICI group. Immune-mediated AEs were infrequent. Emerging data suggesting that ICI treatment might increase subsequent TKI toxicity led us to explore this possibility in our study. We were encouraged that significant toxicity on a subsequent TKI immediately after ICI was infrequent. It is unclear whether there is a direct relationship between ICI and TKI toxicity, but we should remain vigilant.

Our study has several limitations. In addition to its retrospective design and modest sample size, there was heterogeneity in molecular testing methodology, scanning intervals, immunotherapy and chemotherapy regimens, and toxicity details. Our data collection period was before the Food and Drug Administration approvals for *RET*, *MET*, and *KRAS* G12C TKI inhibitors; therefore, the impact of these agents is unknown. We were hoping to provide insight into the role of chemotherapy plus ICI in patients with more rare tumor alterations, but the low numbers of patients precluded this analysis. Importantly, we did not have any patients with *BRAF* V600E mutations and cannot comment on this subgroup. Despite these limitations, this real-world experience aligns with data from ICI monotherapy trials.

In conclusion, our overall results did not reveal a survival benefit for patients with oncogenic-driven tumors with the addition of ICI to chemotherapy, whereas subgroup analyses revealed disparate trends highlighting the heterogeneous composition of the oncogenic driver population suggesting we can no longer group them all together for ICI treatment decision-making. The continued elucidation of immune unresponsiveness is needed to develop effective immunotherapies for this population.

## CRediT Authorship Contribution Statement

**David J. Benjamin:** Investigation, Data curation, Writing—original draft, Writing—review and editing, Visualization.

**Shuai Chen:** Statistical analysis, Methodology, Visualization, Writing—original draft, Writing—review and editing.

**Joanna B. Eldredge:** Investigation, Data curation.

**Shiruyeh Schokrpur:** Investigation, Data curation, Writing—review and editing.

**Debory Li:** Investigation, Data curation.

**Jason W. Chan:** Conceptualization, Methodology, Investigation, Data curation, Writing—review and editing.

**Amy L. Cummings:** Methodology, Resources, Investigation, Data curation, Writing—review and editing, Supervision, Project administration.

**Megan E. Daly:** Conceptualization, Investigation, Writing—review and editing.

**Jonathan W. Goldman:** Conceptualization, Methodology, Investigation, Data curation, Writing—review and editing.

**Matthew A. Gubens:** Resources, Investigation, Writing—review and editing.

**Zhikuan Quan:** Investigation, Data curation, Writing—review and editing.

**Jeremy P. Harris:** Conceptualization, Methodology, Investigation, Writing—review and editing.

**Mark W. Onaitis:** Writing—review and editing.

**Viola W. Zhu:** Conceptualization, Methodology, Investigation, Data curation, Writing—review and editing.

**Sandip P. Patel:** Conceptualization, Methodology, Investigation, Resources, Data curation, Writing—review and editing, Visualization, Supervision, Project administration.

**Karen Kelly:** Conceptualization, Methodology, Investigation, Resources, Data curation, Writing—original draft, Writing—review and editing, Visualization, Supervision, Project administration.
