# Supplementary material for: The Role of Chemotherapy Plus Immune Checkpoint Inhibitors in Oncogenic-Driven NSCLC: A University of California Lung Cancer Consortium Retrospective Study
Source: JTO Clin Res Rep. 2022 Oct 29;3(12):100427. doi: 10.1016/j.jtocrr.2022.100427 (PMC9679033; doi:10.1016/j.jtocrr.2022.100427)

Supplemental Material

Supplemental Table 1: PFS/OS by Smoking, TKI Administration and PD-L1 Expression

Supplemental Table 2: Propensity Score-stratified Survival Analysis for Overall Survival and PFS Using Stratified Cox proportional Hazards Models. Strata were formed by quintiles of estimated propensity scores.

Supplemental Table 3. Grade 3 or Higher Adverse Events with Chemotherapy plus ICI versus Chemotherapy alone

Supplemental Table 4. Grade 3-4 Adverse Event with TKI after chemotherapy plus ICI

Supplemental Figure 1. Study Schema

Supplemental Figure 2: Progression-Free Survival by Subgroups

2A: Progression-Free Survival in Never Smokers

2B: Progression-Free Survival in Smokers

2C: Progression-Free Survival in TKI naïve Patients

2D: Progression-Free Survival in TKI treated Patients

2E: Progression-Free Survival in PD-L1 Negative Tumors

2F: Progression-Free Survival in PD-L1 1-49 Expressing Tumor

2G: Progression-Free Survival in PD-L1 > 50% Expressing Tumor

Supplemental Figure 3: Overall Survival by Subgroups

3A: Overall Survival in Never Smokers

3B: Overall Survival in Smokers

3C: Overall Survival in TKI naïve Patients

3D: Overall Survival in TKI treated Patients

3E: Overall Survival in PD-L1 Negative Tumors

3F: Overall Survival in PD-L1 1-49 Expressing Tumor

3G: Overall Survival in PD-L1 > 50% Expressing Tumor

**Supplemental Table 1: PFS/OS by Smoking, TKI Administration and PD-L1 Expression.**

|  |  | **PFS** | | | **OS** | | |
| --- | --- | --- | --- | --- | --- | --- | --- |
|  | **N** | **HR^a^** | **95%CI**  **(p-value)** | **Median**  **(Days)** | **HR^a^** | **95% CI**  **(p-value)** | **Median**  **(days)** |
| **Never Smokers**  Chemotherapy  Chemotherapy + ICI^b^ | 41  95 | 1.25 | 0.83-1.87  (0.288) | 246  192 | 0.91 | 0.56-1.48  (0.692) | 555  593 |
| **Smokers**  Chemotherapy  Chemotherapy + ICI | 35  75 | 0.98 | 0.63-1.54 | 181  190 | 0.81 | 0.47-1.41 | 464  652 |
| **No TKI**  Chemotherapy  Chemotherapy + ICI | 88  30 | 1.35 | 0.81-2.25 | 289  233 | 1.12 | 0.59-2.15 | NR  1013 |
| **Previous TKI**  Chemotherapy  Chemotherapy + ICI | 46  82 | 1.03 | 0.71-1.51 | 152  172 | 0.80 | 0.51-1.26 | 371  430 |
| **PD-L1 as 0**  Chemotherapy  Chemotherapy + ICI | 23  53 | 1.37 | 0.81-2.34 | 245  145 | 0.96 | 0.51-1.80 | 464  430 |
| **PD-L1 as 1-49**  Chemotherapy  Chemotherapy + ICI | 15  48 | 0.82 | 0.44-1.52 | 111  207 | 0.41 | 0.20-0.83 | 262  653 |
| **PD-L1 as 50+**  Chemotherapy  Chemotherapy + ICI | 13  39 | 1.37 | 0.63-2.99 | 307  225 | 1.00 | 0.39-2.54 | 944  1013 |

^a^ Reported HRs are unadjusted by covariates.

^b^ ICI- immune checkpoint inhibitor

**Supplemental Table 2: Propensity Score-stratified^a^ Survival Analysis for Overall Survival and PFS Using Stratified Cox proportional Hazards Models.**

|  | **PFS** | | | **OS** | | |
| --- | --- | --- | --- | --- | --- | --- |
|  | **HR^b^** | **(95% CI)** | **p-value** | **HR^b^** | **(95% CI)** | **p-value** |
| **All Patients**  Treatment  Chemotherapy  Chemotherapy+ ICI^c^ | 1.00  1.29 | 0.87-1.90 | 0.198 | 1.00  0.94 | 0.60-1.48 | 0.797 |
| **Subset of Oncogenic Driven EGFR**  Treatment  Chemotherapy  Chemotherapy + ICI | 1.00  1.67 | 1.00-2.80 | 0.049 | 1.00  1.14 | 0.64-2.04 | 0.659 |
| **Subset of Oncogenic Driven KRAS**  Treatment  Chemotherapy  Chemotherapy + ICI | 1.00  0.66 | 0.29-1.50 | 0.327 | 1.00  0.42 | 0.16-1.08 | 0.071 |
| **Subset of non-EGFR/KRAS Oncogenic Driven**  Treatment  Chemotherapy  Chemotherapy + ICI | 1.00  1.67 | 0.44-6.25 | 0.450 | 1.00  0.63 | 0.14-2.74 | 0.538 |
| **Subset of Oncogenic Driven KRAS G12C**  Treatment  Chemotherapy  Chemotherapy + ICI | 1.00  0.31 | 0.07-1.46 | 0.140 | 1.00  0.41 | 0.08-2.06 | 0.277 |
| **Subset of Smoker**  Treatment  Chemotherapy  Chemotherapy + ICI | 1.00  1.06 | 0.59-1.90 | 0.851 | 1.00  0.73 | 0.36-1.51 | 0.398 |
| **Subset of Non-Smoker**  Treatment  Chemotherapy  Chemotherapy + ICI | 1.00  1.52 | 0.86-2.67 | 0.150 | 1.00  1.02 | 0.56-1.86 | 0.947 |
| **Subset of Receiving chemo or chemo IO as First Line**  Treatment  Chemotherapy  Chemotherapy + ICI | 1.00  1.69 | 0.84-3.38 | 0.138 | 1.00  0.97 | 0.43-2.16 | 0.932 |
| **Subset of Receiving chemo or chemo IO beyond First Line**  Treatment  Chemotherapy  Chemotherapy + ICI | 1.00  1.20 | 0.74-1.96 | 0.455 | 1.00  0.92 | 0.53-1.60 | 0.777 |
| **Subset of PD-L1 as 0**  Treatment  Chemotherapy  Chemotherapy + ICI | 1.00  1.57 | 0.88-2.80 | 0.124 | 1.00  0.98 | 0.50-1.90 | 0.949 |
| **Subset of PD-L1 as 1-49**  Treatment  Chemotherapy  Chemotherapy + IOI | 1.00  1.08 | 0.52-2.26 | 0.838 | 1.00  0.70 | 0.29-1.69 | 0.430 |
| **Subset of PD-L1 as 50+**  Treatment  Chemotherapy  Chemotherapy + ICI | 1.00  1.99 | 0.78-5.09 | 0.149 | 1.00  1.65 | 0.61-4.47 | 0.323 |

^a^ Strata were formed by quintiles of estimated propensity scores.

^b^ Reported HRs are adjusted by covariates using propensity score method.

^c^ ICI-Immune checkpoint inhibitor

**Supplemental Table 3. Grade 3 or Higher Adverse Events with Chemotherapy plus ICI versus Chemotherapy alone**

| **Adverse Event** | **Chemotherapy and ICI**  **N= 176** | **Chemotherapy**  **N=70** |
| --- | --- | --- |
| Total Number of Events | 30 (17.0%) | 33 (47.1%) |
| Neutropenia | 2 (1.1%) | 6 (8.6%) |
| Nausea/vomiting | 0 (0%) | 2 (2.9%) |
| Thrombocytopenia | 3 (1.7%) | 6 (8.6%) |
| Mucositis | 1 (0.57%) | 2 (2.9%) |
| Fatigue | 3 (1.7%) | 1 (1.4%) |
| Diarrhea | 1 (0.57%) | 0 (0%) |
| Dehydration | 1 (0.57%) | 1 (1.4%) |
| Anorexia | 1 (0.57%) | 1 (1.4%) |
| Failure to thrive | 0 (0%) | 1 (1.4%) |
| Cellulitis | 1 (0.57%) | 1 (1.4%) |
| Acute kidney injury | 0 (0%) | 1 (1.4%) |
| Neutropenic fever | 1 (0.57%) | 1 (1.4%) |
| Hyponatremia | 1 (0.57%) | 0 (0%) |
| Myocarditis | 1 (0.57%) | 0 (0%) |
| Pneumonitis | 2 (1.1%) | 0 (0%) |
| Rash | 3 (1.7%) | 1 (1.4%) |
| Transaminitis | 1 (0.57%) | 0 (0%) |
| Hypertension | 1 (0.57%) | 0 (0%) |
| Hyperthyroidism | 1 (0.57%) | 0 (0%) |
| Hypotension and syncope | 0 (0%) | 1 (1.4%) |
| Tongue swelling | 1 (0%) | 0 (0%) |
| Anemia | 4 (2.3%) | 5 (7.1%) |
| Pulmonary embolism | 0 (0%) | 1 (1.4%) |
| Neuropathy | 0 (0%) | 1 (1.4%) |
| Orthostatic hypotension | 0 (0%) | 1 (1.4%) |
| Nephritis | 1 (0.57%) | 0 (0%) |

**Supplemental Table 4. Grade 3-4 Adverse Event with TKI after chemotherapy plus ICI**

| **Adverse Event** | **Number of Events** | **TKI** | **Time from TKI** | **Mutation** |
| --- | --- | --- | --- | --- |
| Transaminitis | 5 | Alectinib  Crizotinib  Adagrasib  Osimertinib plus alisertib  Osimertinb | 23 days  27 days  57 days  101 days  103 days | EML4-ALK  BRAF G466V  KRAS G12C  EGFR exon 19 deletion  EGFR L858R/EGFR exon 19 deletion |
| Hypersensitivity | 1 | Selpercatinib | 8 days | RET-KIF5B fusion |
| Thrombocytopenia | 1 | Selpercatinib | 8 days | RET-KIF5B fusion |
| Hemolytic anemia | 1 | Alectinib | 23 days | EML4-ALK |
| Steven-Johnsons Syndrome | 1 | Osimertinib | 32 days | EGFR L858R |
| Hyponatremia | 1 | Osimertinib plus alisertib | 101 days | EGFR exon 19 deletion |

**Supplemental Figure 1. Study Schema**

Eligibility Criteria

a) Stage IV NSCLC

b) Actionable Mutation

(EGFR, ALK, ROS1,

MET, RET, HER2,

KRAS, BRAF, NTRK)

identified by a CLIA certified

laboratory

c) Received platinum-based

chemotherapy with or without

an immune checkpoint inhibitor

(ICI) in 2018-2019

Chemotherapy cohort N= 76

Actionable Mutation

EGFR 49 patients (64.5%)

KRAS 15 patients (19.7%)

ALK 6 patients (7.9%)

HER2 2 patients (2.6%)

ROS1 2 patients (2.6%)

MET 1 patient (1.3%)

BRAF 1 patient (1.3%)

(non-V600E)

Chemotherapy + ICI N=170

Actionable Mutation

EGFR 86patients (50.6%)

KRAS 66 patients (38.8%)

ALK 7 patients (4.1%)

HER2 5 patients (2.9%)

RET 2 patients (1.2%)

MET 2 patients (1.2%)

ROS1 1 patient (0.6%)

BRAF 1 patient (0.6%)

(non-V600E)

**Supplemental Figure 2 Progression-Free Survival by Subgroups**

**Figure 2A. Never Smokers**


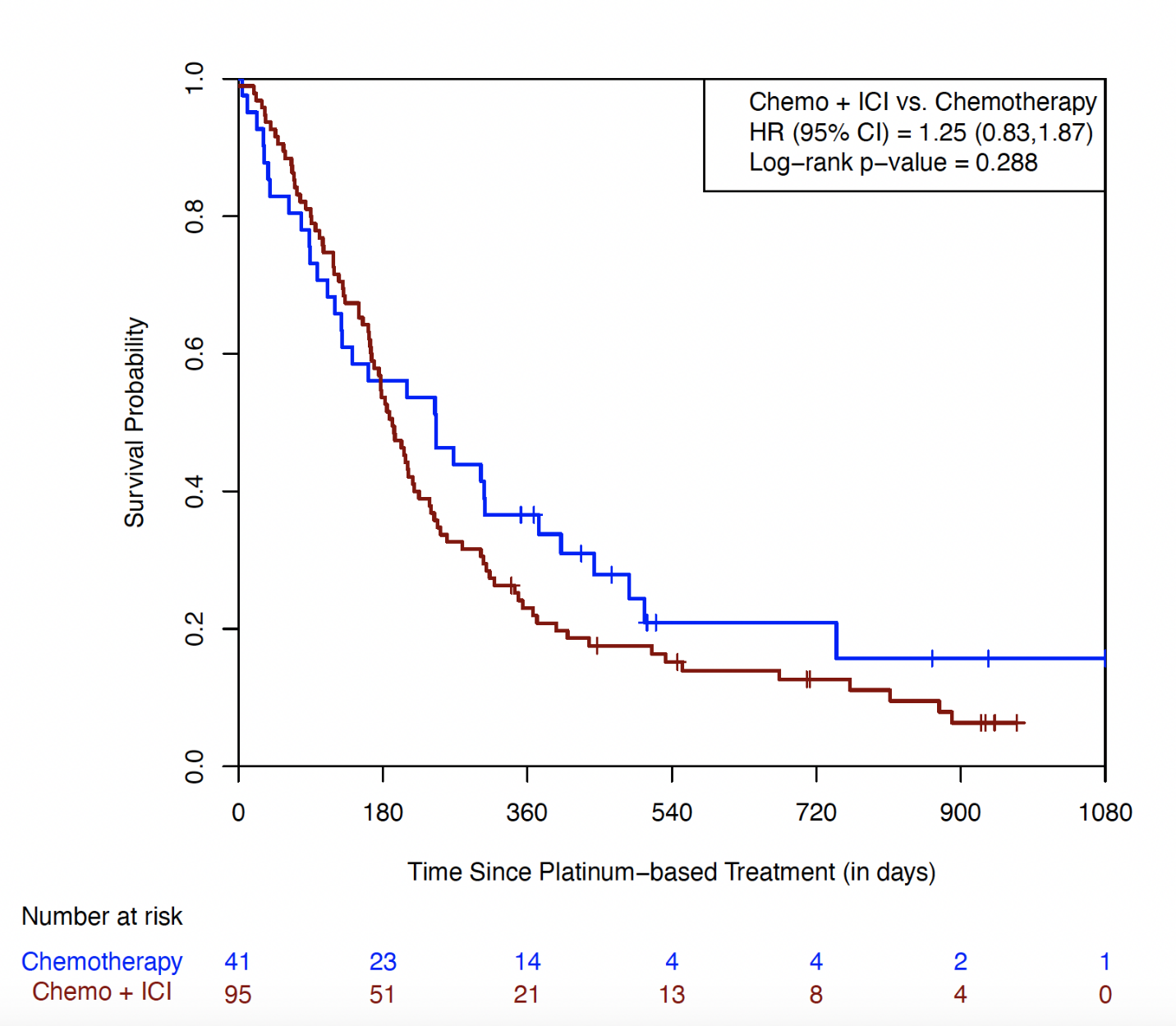


**Supplemental Figure 2B.** **Smokers**


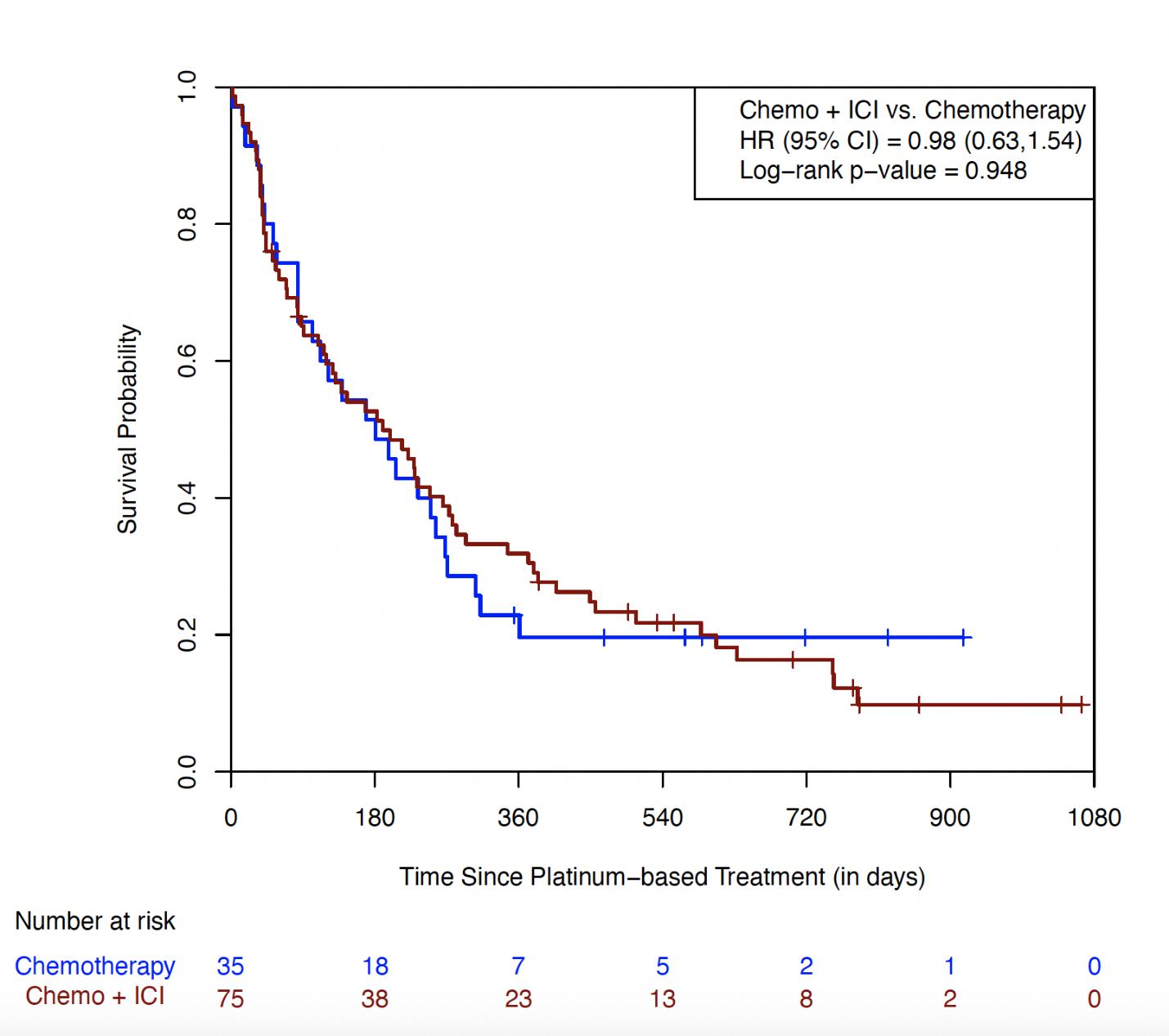


**Supplemental Figure 2C. TKI Naïve Patients**


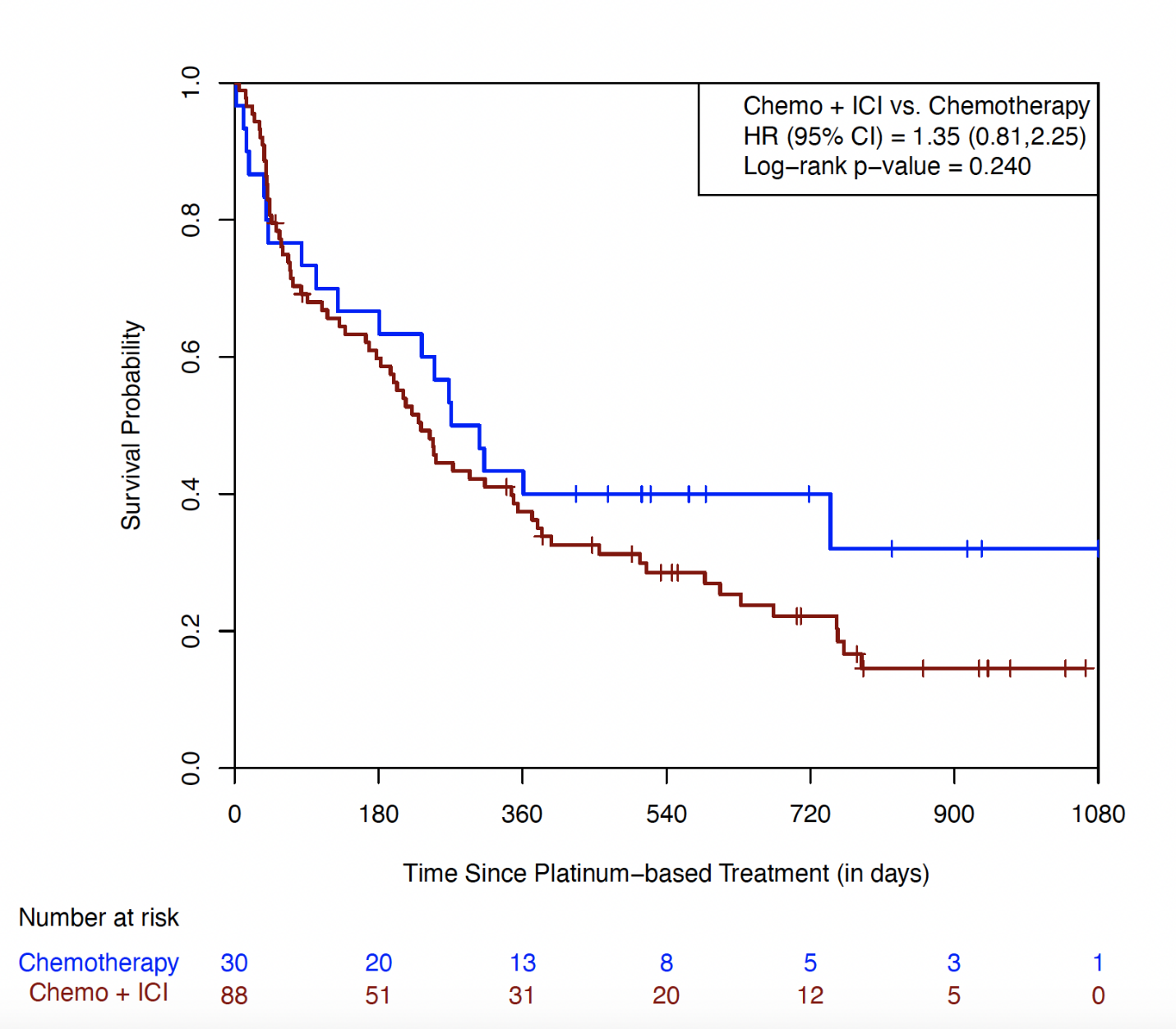


**Supplemental Figure 2D. TKI-treated Patients**


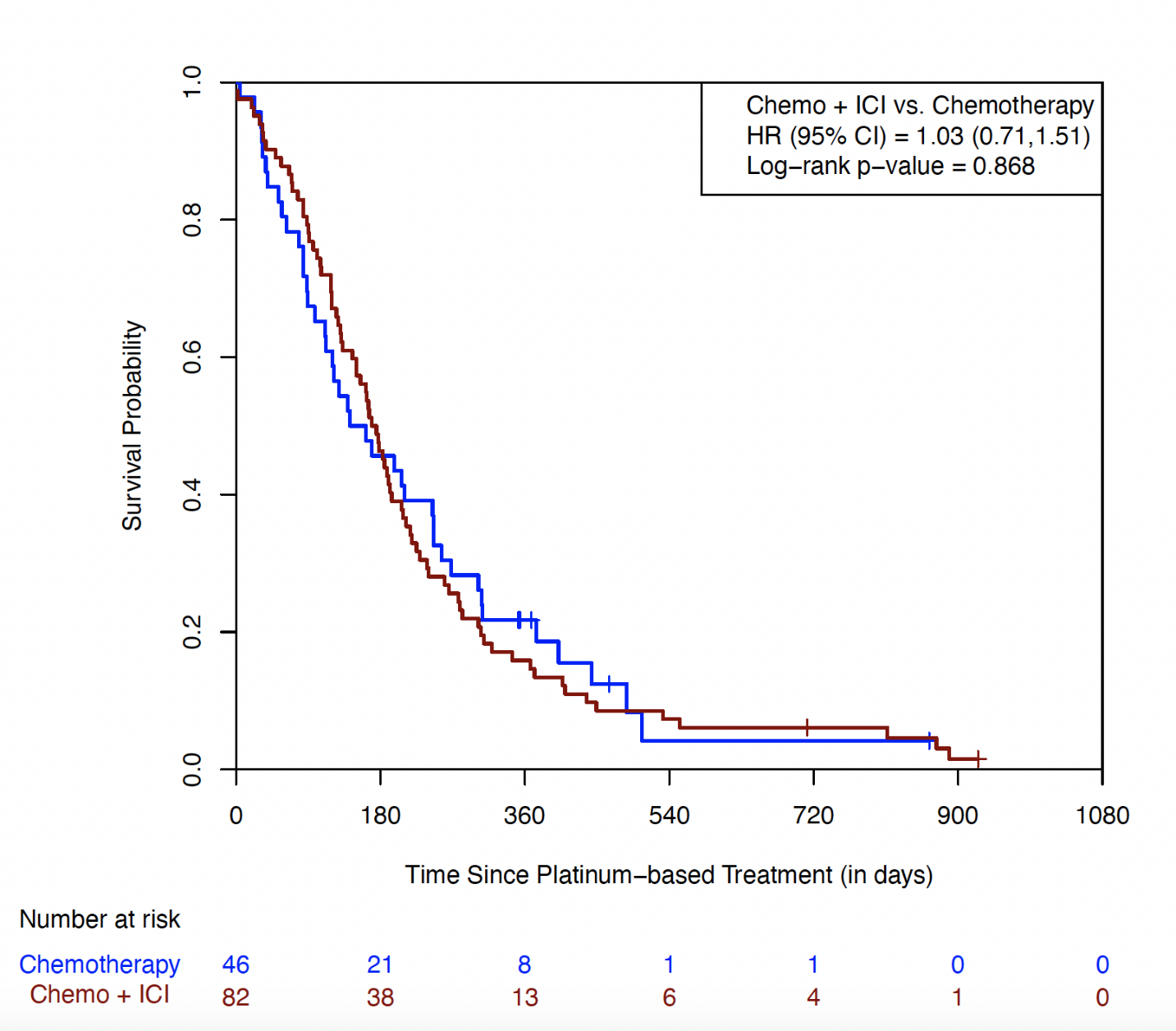


**Supplemental Figure 2E. PD-L1 Negative Tumors**


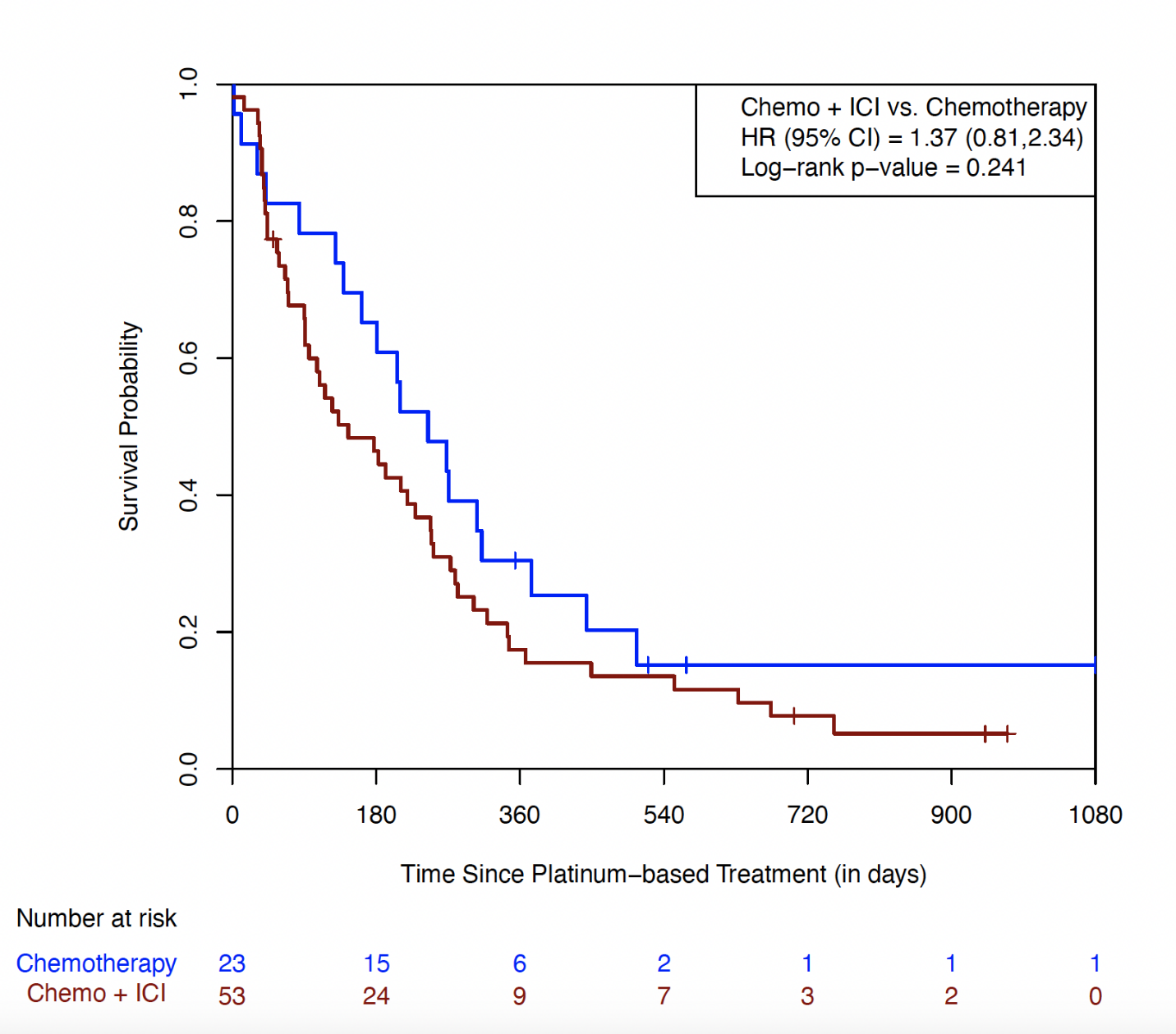


**Supplemental Figure 2F. PD-L1 1-49% Expressing Tumors**


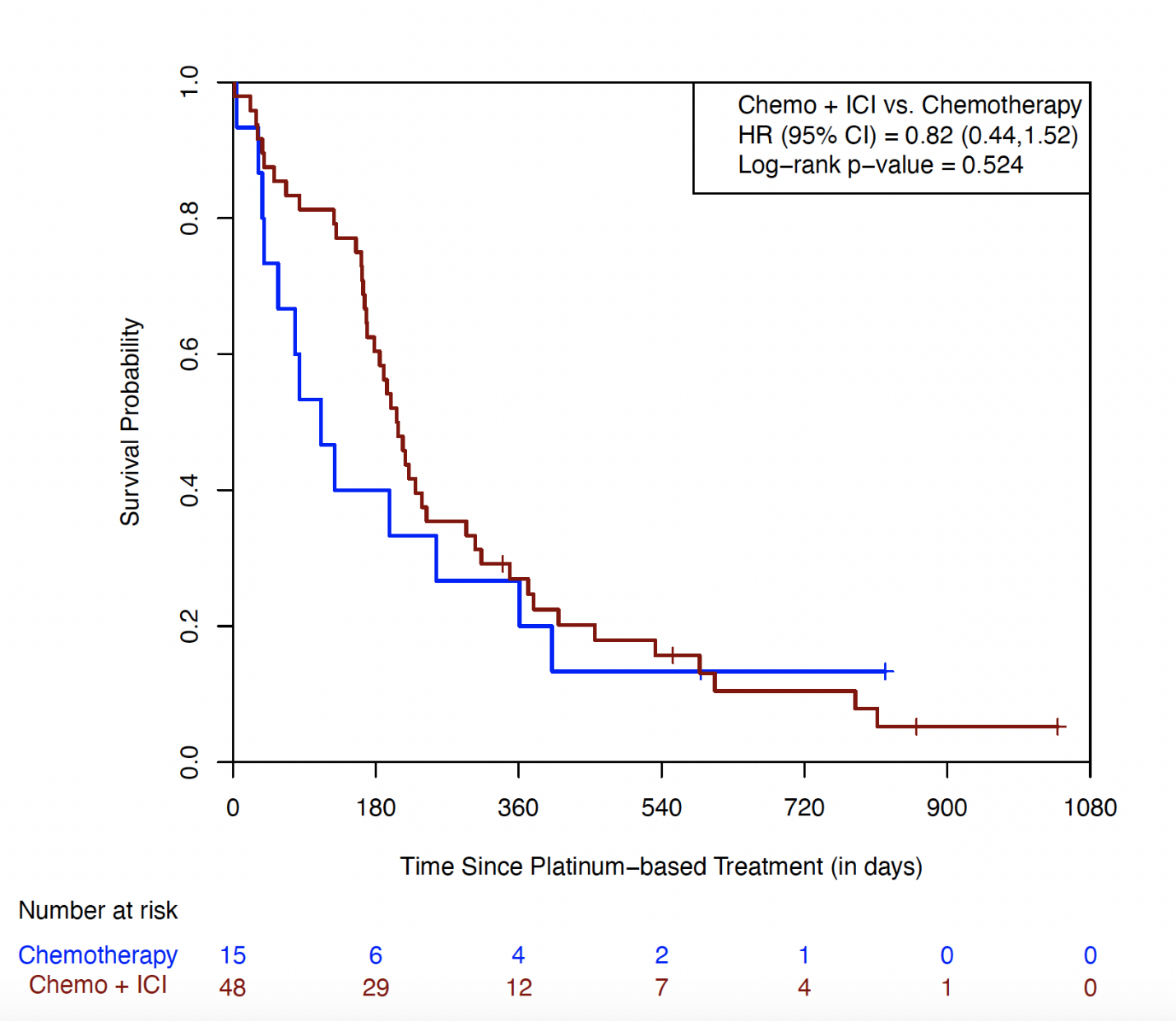


**Supplemental Figure 2G. PD-L1 > 50% Expressing Tumors**


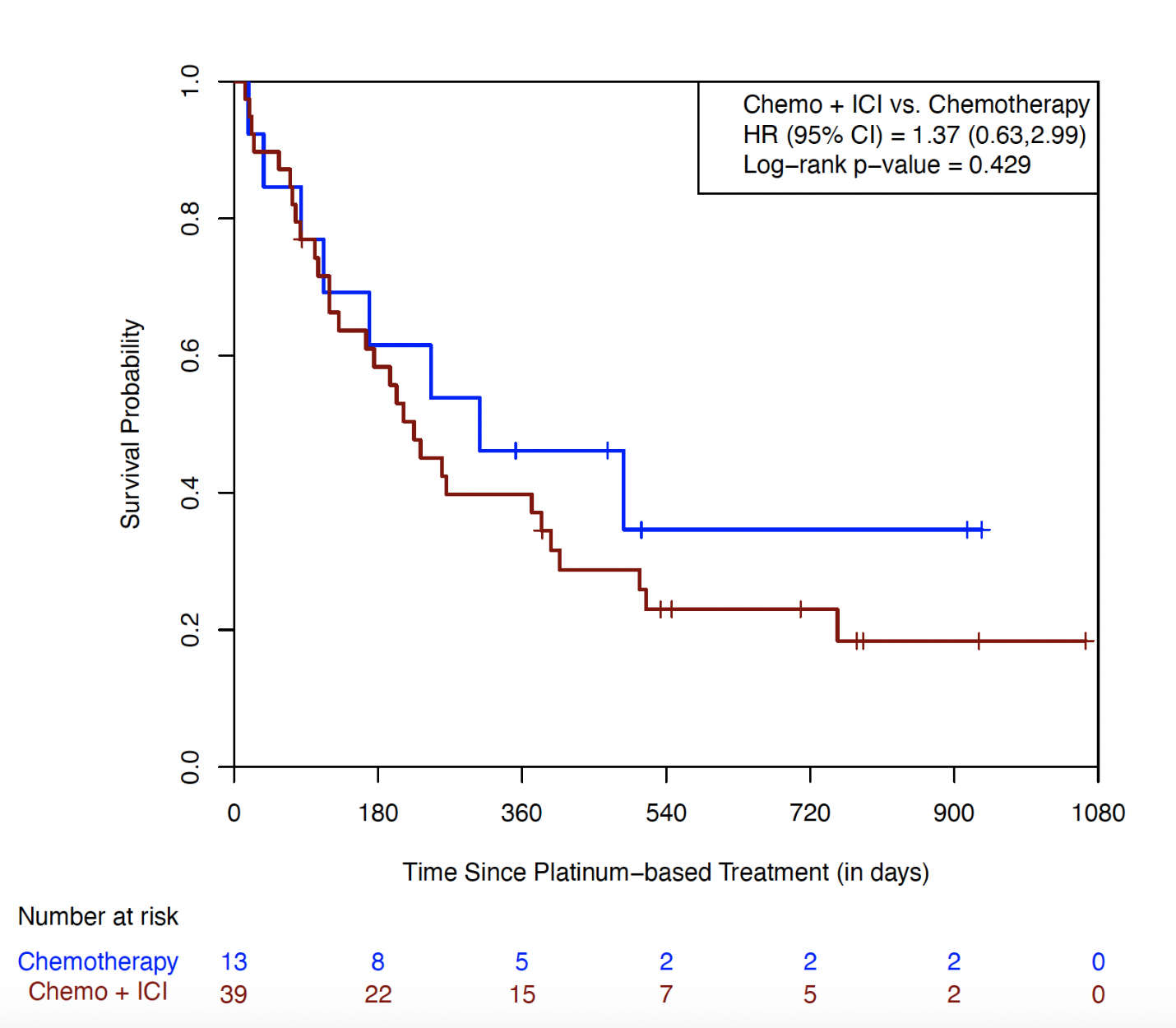


**Supplemental Figure 3. Overall Survival by Subgroups**

**Figure 3A. Never Smokers**


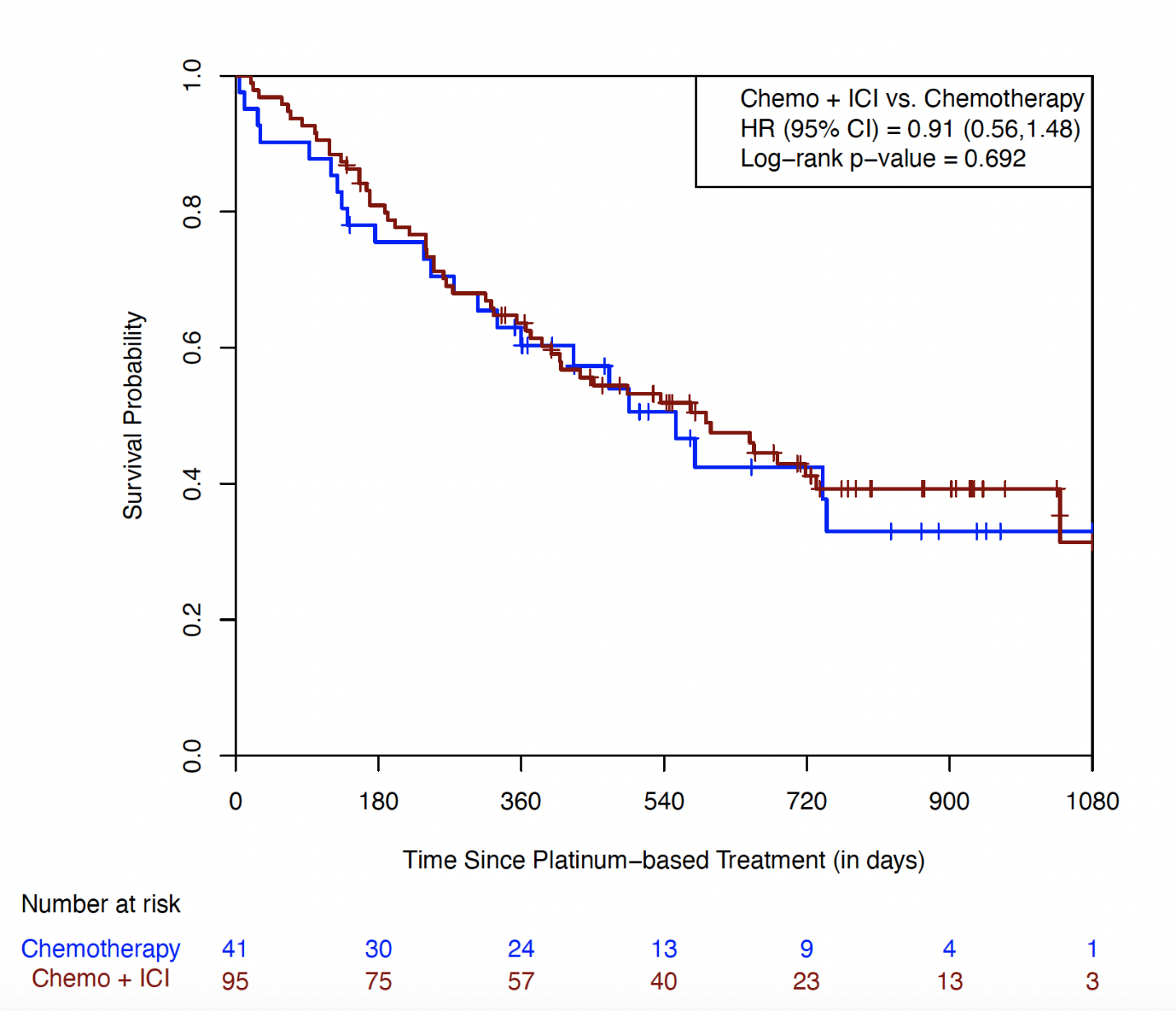


**Supplemental Figure 3B. Smokers**


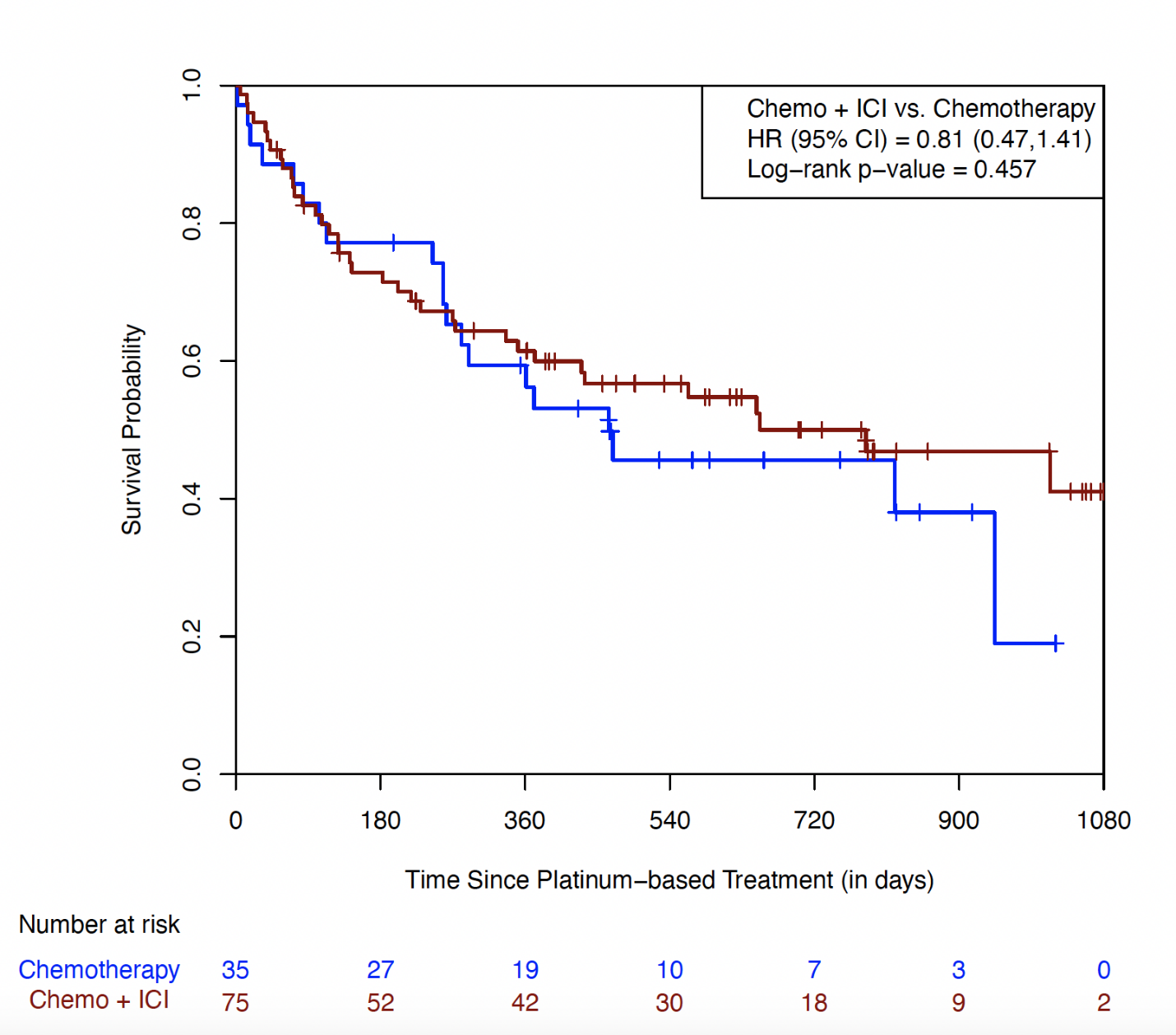


**Supplemental Figure 3C. TKI-Treatment Naïve Patients**


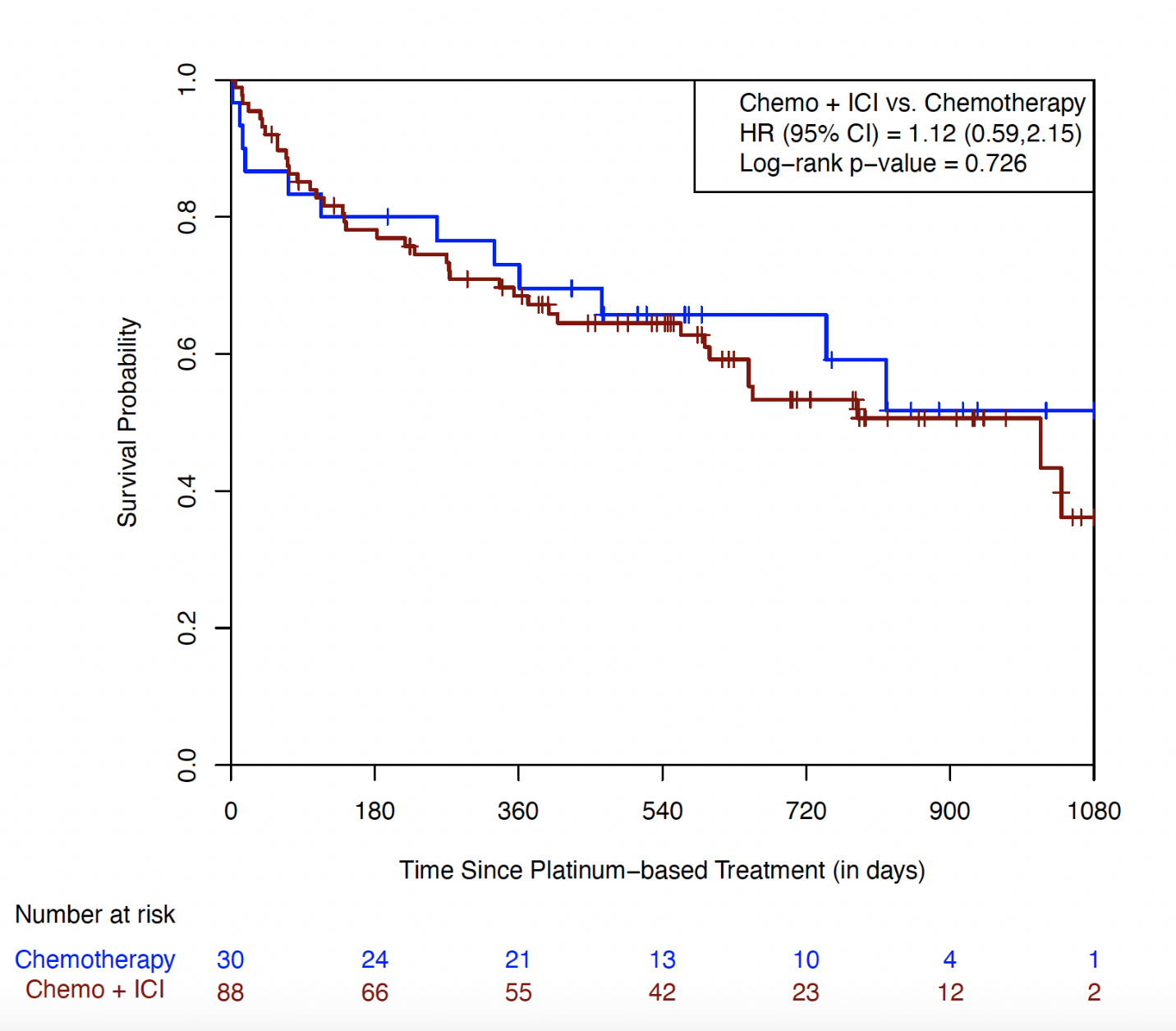


**Supplemental Figure 3D. TKI-treated Patients**


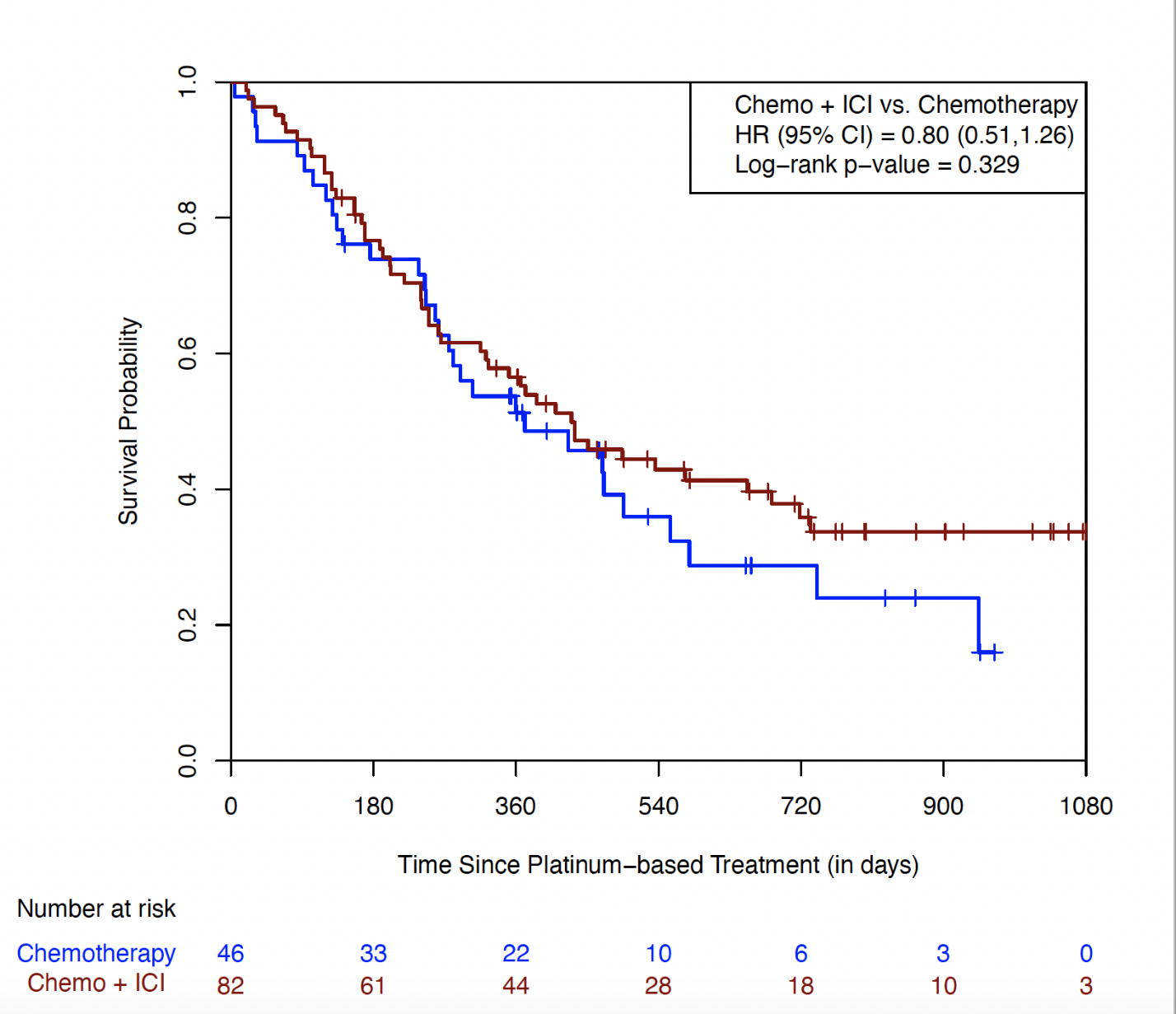


**Supplemental Figure 3E. PD-L1 Negative Tumors**


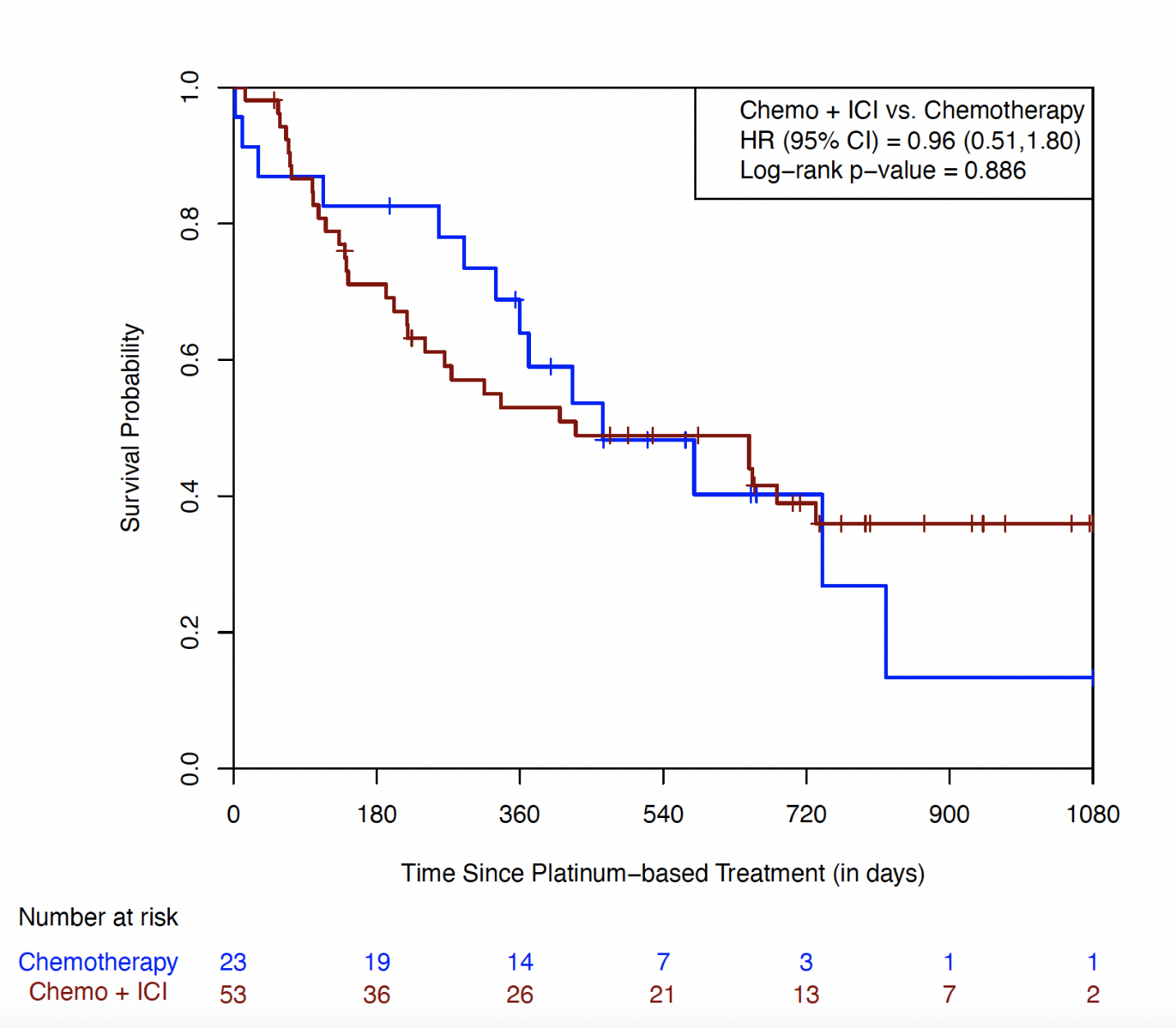


**Supplemental Figure 3F. PD-L1 1-49% Expressing Tumors**


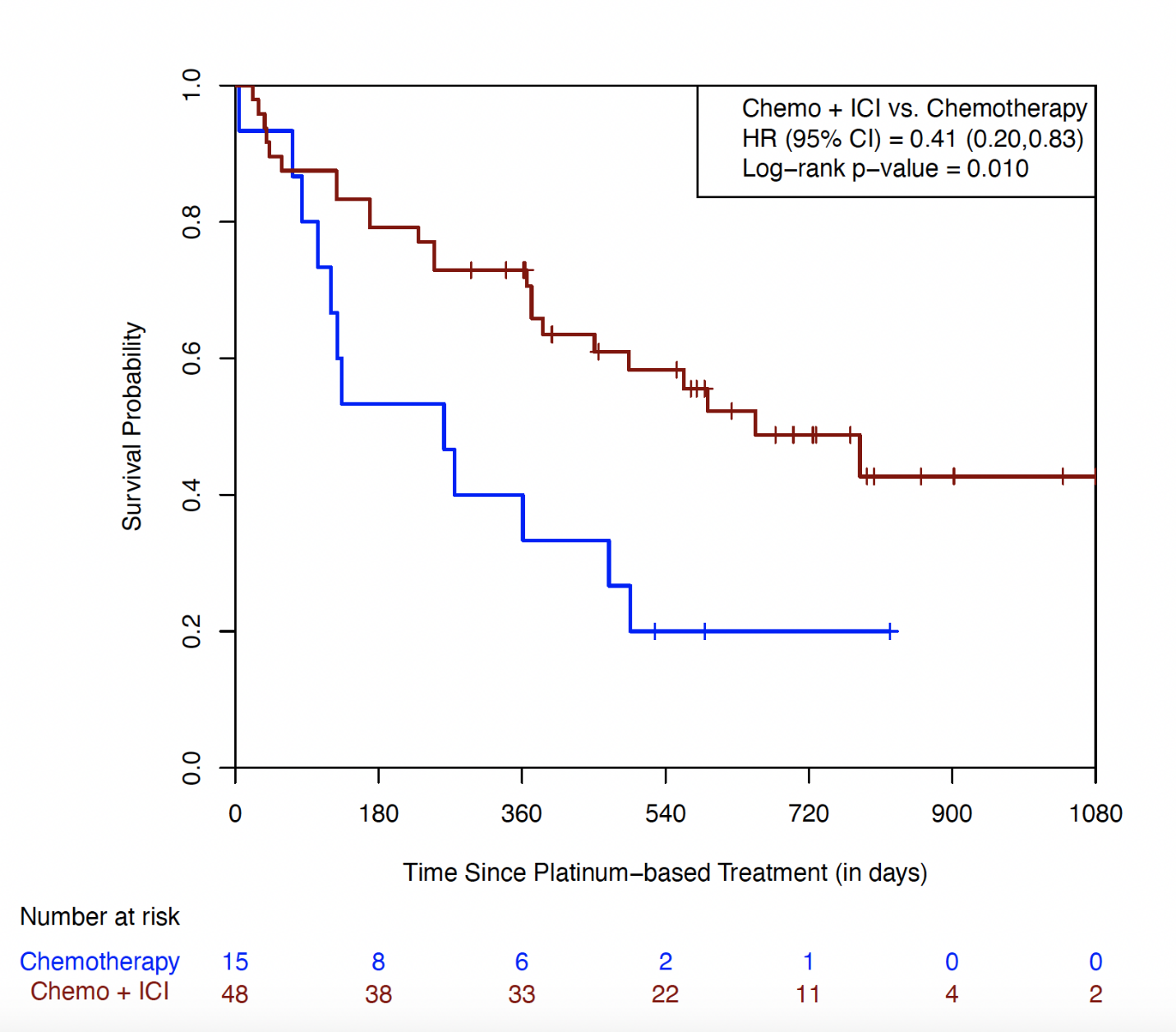


**Supplemental Figure 3G. PD-L1 > 50% Expressing Tumors**


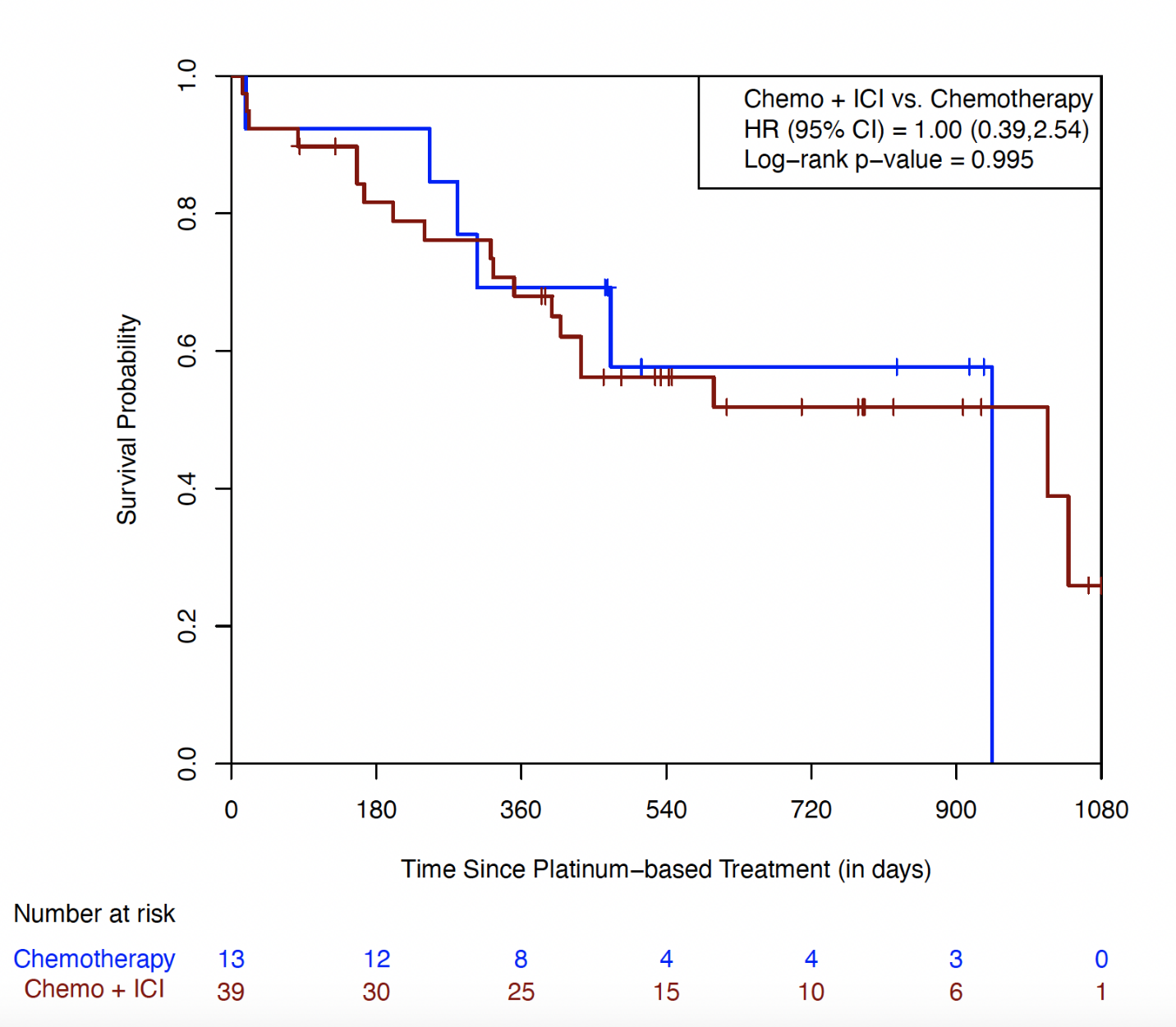

Supplement: Supplemental Tables 1-4, Figures 1 and 2 [file mmc1.docx]
